# Spatiotemporal dynamics and adaptive strategies of plant diversity and functional traits in desert steppe

**DOI:** 10.3389/fpls.2026.1763260

**Published:** 2026-04-17

**Authors:** Shiya He, Tiantian Wu, Yanxin Yang, Shutong Yang, Weihong Hou, Xuhui Tang, Adilamu Abulaiti, Xiaofang Ye, Fei Yu, Lingxiu Wang, Jie Wang, Huixia Liu

**Affiliations:** 1 School of Life Sciences, Xinjiang Normal University, Urumqi, China; 2 International Center for the Collaborative Management of Cross-border Pests in Central Asia, Urumqi, China; 3 Tacheng, Research Field (Migratory Biology), Observation and Research Station of Xinjiang, Tacheng, China; 4 Urumqi Meteorological Bureau, Urumqi Pastoral Meteorological Experimental Station, Central Tianshan Grassland Ecology Monitoring Laboratory, Urumqi, Xinjiang, China

**Keywords:** desert steppe, functional traits, Ili river basin, plant diversity, spatiotemporal dynamics

## Abstract

**Introduction:**

Plant diversity and functional traits form the core foundation of grassland ecosystem stability. However, rapid climate change poses a severe threat to biodiversity, making it imperative to clarify how these two factors mediate community responses to environmental changes across spatiotemporal scales.

**Methods:**

By integrating field surveys with laboratory analyses, we have investigated the spatiotemporal patterns of plant diversity and key functional traits within desert grasslands of the Ili River Basin.

**Results:**

We found that: (1) Plant diversity in May and July was significantly higher than in September, with distinct seasonal dynamics observed across different plant community types; (2) During the main growing season, plant functional traits exhibited a marked increasing trend along the elevation gradient; (3) Both plant functional traits and diversity displayed a patchy mosaic distribution pattern, accompanied by significant spatial heterogeneity. High diversity values were predominantly located around Nilek and Huocheng counties, while low-value areas were distributed in Xinyuan and Gongliu counties. High-value areas for plant functional traits were concentrated in Nilek county, with low-value areas found in Xinyuan, Gongliu, and Huocheng counties. (4) Redundancy Analysis (RDA) and Structural Equation Modeling (SEM) indicate that soil moisture content and soil nitrogen are the primary drivers of diversity variation, while soil carbon-to-nitrogen ratio and precipitation regulate functional trait variation.

**Discussion:**

These findings demonstrate how plants adapt to environmental heterogeneity through coordinated changes in diversity and functional traits, offering a scientific basis for conserving and managing desert steppe ecosystems.

## Graphical Abstract

**Figure:**
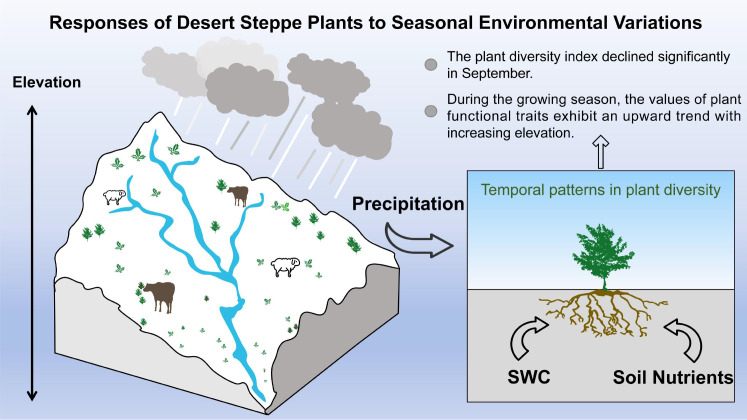

## 1 Introduction

Plant diversity is a cornerstone of ecosystem structure and function, serving as a key indicator of community composition while supporting resilience and productivity (Craven et al., 2018). Plant functional traits serve as pivotal indicators of resource allocation strategies, profoundly elucidating the adaptive trade-off mechanisms through which plants respond to environmental change. They represent the integrated manifestation of morphological and physiological characteristics in environmental adaptation (Funk et al., 2017; Jäschke et al., 2020; Wright et al., 2004). Together, they are indispensable for predicting and assessing ecosystem stability (Cadotte, 2017; Hu et al., 2022; Huxley et al., 2023; Chen et al., 2025a). Current research, however, has primarily focused on temperate, tropical, and alpine regions, with studies on other ecosystems, particularly desert steppes, remaining limited. Existing literature often follows two relatively independent paths: one examines the drivers of plant diversity, such as precipitation (Zhang et al., 2021b), elevation (He et al., 2023a), and soil properties (Laliberté et al., 2013; Jiang et al., 2022), and their role in ecosystem stability (Gulzar et al., 2024; Wang et al., 2025b); The other examines plant functional trait responses to environmental gradients like elevation (Hu et al., 2024), soil nutrients (Li et al., 2022; Feng et al., 2024), and grazing disturbance (Ye et al., 2020). While these perspectives have advanced our understanding, the intrinsic linkages and synergistic mechanisms between plant diversity and functional traits under environmental change remain unclear. Critically, diversity and traits likely do not operate in isolation but drive ecosystem outcomes through synergies and trade-offs (Ouyang et al., 2023). Therefore, elucidating how they co-vary to mediate adaptation is crucial for advancing ecosystem predictions and supporting the restoration of degraded systems.

Under the dual pressures of accelerating climate change and human activities, grassland ecosystems are confronting escalating threats, most notably biodiversity loss, functional degradation, and desertification (Cheng et al., 2021). As an integration of climatic and edaphic drivers, vegetation dynamics play a pivotal role in maintaining ecosystem balance and underpinning sustainable development (Lehnert et al., 2015; Thakur et al., 2021). In desert steppes, plants have evolved pronounced seasonal life-history strategies, rendering the study of seasonal dynamics essential for predicting how diversity and traits respond to biotic and abiotic fluctuations (Facelli et al., 2005; Al Hajj et al., 2024). To bridge the gap between individual-level adaptations and ecosystem-scale processes, the Community-Weighted Mean (CWM) of functional traits serves as a robust metric. By scaling the Leaf Economics Spectrum (LES) from individual species to the community level, the CWM approach allows more accurately capture the aggregate resource-use strategies and their implications for ecosystem stability in response to environmental filtering (Luo et al., 2023; Bello et al., 2021). Consequently, a synergistic investigation that aligns plant diversity with functional traits across seasons is vital for deciphering the complex processes governing desert steppe ecosystem. How do plant diversity and functional traits co-vary throughout the growing season, and how do different plant communities vary in their adaptive response?

To address these questions, an integrated analysis within a seasonal framework is needed. Furthermore, a complete understanding requires connecting temporal dynamics with spatial patterns. Plant community composition and function are not uniformly distributed. Their spatial heterogeneity is a critical manifestation of ecosystem structure and function (Barbolini et al., 2020; Song et al., 2025). Geographic Information Systems (GIS) are powerful tools for visualizing and quantifying these spatial patterns (Pereira et al., 2021), which link plot-scale measurements with broader topographic, soil, and microclimatic gradients. Adopting a spatial perspective alongside temporal monitoring reveals otherwise elusive ecological patterns (Qiu et al., 2025). These patterns clarify seasonal changes in plant diversity and functional traits, providing a better basis for predicting ecosystem dynamics.

As a pivotal grassland transition zone (Dong et al., 2010), the desert steppe is characterized by simple structure, ecological fragility, and low stability (Pan et al., 2024). It serves crucial economic functions, such as providing seasonal pasture, and performs vital ecological roles, including wind erosion control, sand fixation, and soil and water conservation (Hou et al., 2021; Bai and Cotrufo, 2022; Li et al., 2025a). This region is highly sensitive to climate fluctuations and human activities, impacting regional ecological security (Ohlert et al., 2025; Wang et al., 2026). To understand how plant communities in this fragile system adapt to spatiotemporal environmental variations, the Leaf Economics Spectrum (LES) provides a powerful theoretical lens. It posits that plant functional traits are coordinated along a continuous axis from “fast” resource acquisition to “slow” resource conservation strategies, directly linking individual plant adaptations to ecosystem functioning. Given that the elevational gradient of the Ili River Basin’s desert steppe is accompanied by significant redistribution of water, heat, and soil resources. Typically, although increasing elevation leads to lower temperatures, during the peak growing season (July), snowmelt and increased precipitation collectively enhance soil water availability in higher-elevation areas, effectively mitigating drought stress. Concurrently, the study area hosts numerous annual plant species, which require rapid nutrient accumulation during their growth peak to store resources for critical life history stages. Based on Leaf Economics Spectrum (LES) theory and the aforementioned regional characteristics, we propose the following core hypotheses: H1 (Strategic Shift under Elevational Gradient): During July, when water stress is alleviated, plants at higher elevations will temporarily deviate from the LES-predicted “resource-conservative” norm, adopting instead a “rapid acquisition” strategy (manifested as increased leaf area and elevated nutrient content). This enables them to fully use the limited growing season window to complete their life history. H2 (Strategic Transition Along Seasonal Gradient): Plant resource strategies dynamically shift throughout the growing season. May (re-greening period) prioritizes breaking dormancy and initiating growth; July reaches peak resource acquisition under optimal thermohygric conditions; September (wilting period) transitions to resource storage and stress resistance, reverting to the “slow-conservative” strategy described by LES. Therefore, grounded in the LES framework, this study investigates the desert steppe of the Ili River Basin through vegetation surveys in May, July, and September, to address three key questions: (1) How does plant diversity across plant communities vary seasonally? (2) What are the spatiotemporal patterns of plant functional traits, as key dimensions of the LES, along elevational gradients? (3) How does the spatial heterogeneity of plant diversity and functional traits respond to environmental filtering, and how does this filtering shape the observed LES strategy spectrum? By clarifying the synergistic mechanisms and spatiotemporal dynamics among species composition, diversity, and traits, this study aims to provide a theoretical foundation for protecting and sustaining these ecologically sensitive regions and for formulating effective management strategies.

## 2 Materials and methods

### 2.1 Study area

**Figure 1:**
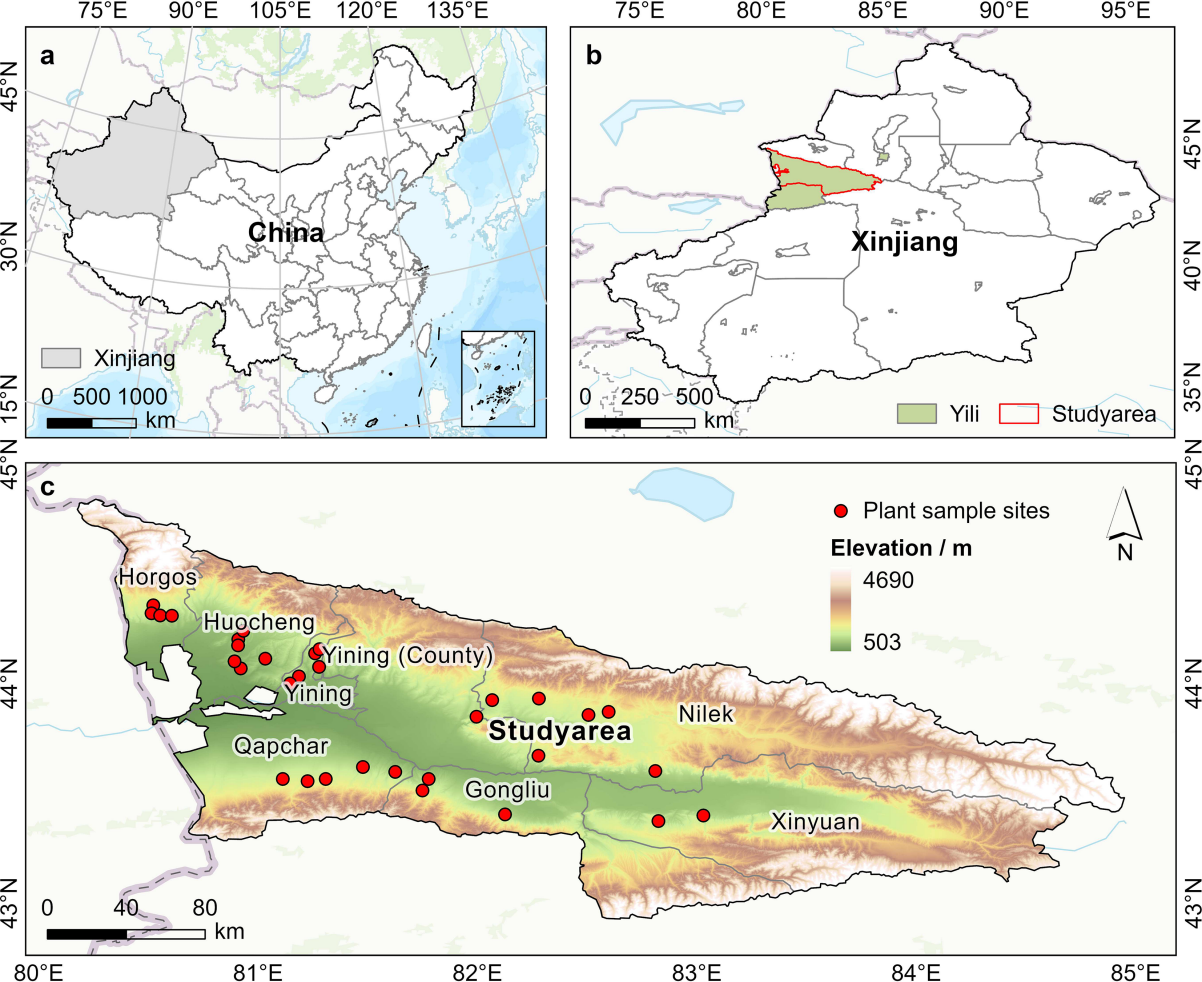
The study area and distribution of plant sampling sites. **(A)** China; **(B)** Xinjiang; and **(C)** Study area. Digital elevation model (DEM) of the study area, illustrating the spatial distribution of plant sampling sites (red dots). Visualized based on the standard map (Review Number: GS(2024)0650) obtained from the National Geographic Information Public Service Platform of China.

The study area is situated within the desert steppe of the Ili River basin in the western Tianshan Mountains, with geographical coordinates ranging from 80°48’–91°39’E and 42°83’–44°39’N (**Figure 1**). The climate of this region is typically temperate continental, with an average elevation of 1063.53 m. The average annual precipitation is about 371 mm, and the average annual temperature is between 6.2 –9.7 °C. The predominant soil type is grey desert soil. The study area hosts diverse dominant herbaceous plant communities, chiefly comprising *Seriphidium transiliense*, *Ceratocarpus arenarius, Carex liparocarpos, Elymus nutans, Sophora alopecuroides, and Peganum harmala.*

### 2.2 Plot design

Between May and September 2024, we systematically established 32 standardized sampling plots (100 m × 100 m) on desert grasslands within the Ili River Basin. The selection of sampling periods is based on the climatic characteristics and vegetation phenology patterns of the study area. Precipitation in this region exhibits a typical pulsed pattern: late spring to early summer represents a relatively concentrated precipitation period, with minor precipitation events predominating. Occasional short-duration heavy rainfall events during summer (particularly July) can replenish deep soil moisture. Vegetation phenology exhibits the following patterns: vegetation emerges from late March to April, enters the initial growth phase in May, reaches peak growth in July, and from September onwards, annual plants progressively wither and turn yellow while perennial plants gradually enter dormancy (Huang et al., 2025; Li et al., 2025b). Based on these phenological characteristics and the precipitation pulse pattern, this study selected May, July, and September for sampling to comprehensively capture the dynamic responses of plant traits to varying seasonal moisture conditions and growth stages. It should be noted that July represents the peak growing season for plants, when foliage development is most complete and functional traits are expressed with the greatest stability, thereby providing the most accurate reflection of community functional structure characteristics. Therefore, during July sampling, we supplemented the original 32 permanent plots with additional temporary sampling points along the elevation gradient. This expanded coverage encompassed a more comprehensive elevation range (750–2500 m), enabling capture of the core response patterns of community functional traits along the elevation gradient. Sampling in May and September was conducted within the original 32 permanent plots. **Supplementary Material** provides a spatial distribution map of the July sampling points (**Supplementary Figure 8**), visually illustrating the spatial coverage of sampling during this month. Using the grid method, establish five 1-meter × 1-meter herbaceous plots within each large sample plot. During each survey, record all visible and identifiable plant individuals within the sample plot. For perennial plants, if their aboveground parts have completely withered or disappeared (such as during the dormant period in September), they shall not be included in the species record for that survey. This criterion ensures that each survey reflects the active species composition within the community at that time. Based on the importance values of species within each plot, all species were ranked from highest to lowest importance value. The family to which the species with the highest importance value belonged was identified as the dominant family for that plot (Equation 1). Subsequently, based on the dominant family, all plots in the study area were classified into six dominant family-dominated community types: Asteraceae-dominated, Poaceae-dominated, Cyperaceae-dominated, Fabaceae-dominated, Chenopodiaceae-dominated, and other families-dominated types. Community composition results indicate: The Poaceae community was dominated by *Elymus nutans* and *Stipa capillata*, while Asteraceae communities are represented by *Seriphidium transiliense.* Chenopodiaceae-dominated communities feature *Ceratocarpus arenarius* as the dominant species, while the Cyperaceae community was dominated by *Carex liparocarpos*; The Fabaceae community was primarily composed of *Sophora alopecuroides* and *Trigonella arcuata*, with other families represented by species such as *Grubovia dasyphylla.*

### 2.3 Selection of species diversity

1. Plant height was determined using a combination of tape measure measurements and visual estimation. Within each plot, standard plants exhibiting good growth and free from disease, pests, or mechanical damage were selected. Their standing height from ground level to the highest vegetative organ was measured using a steel tape measure accurate to 0.1 cm (Halbritter et al., 2024). Plant density surveys were conducted concurrently within the aforementioned plots using the species-by-species counting method (Cottam and Curtis, 1956). Plant cover was defined as the percentage of plot area occupied by the vertical projection of aboveground plant parts. Biomass was obtained by harvesting the aboveground parts of each plant species at ground level and weighing their fresh mass. For laboratory analysis, returned plant samples were dried in an 80 °C oven for 48 hours to constant weight, then weighed to determine dry mass (Dawson et al., 2022).

2. Importance value (*IV*) (Li et al., 2024) was used to show the dominant species in the steppe-desert community.

(1) $$IV=\frac{RD+RH+RC+RB}{4}$$

RD, RH, RC, and RB are the relative density, relative height, relative cover and relative above-ground biomass.

3. We selected four commonly used diversity indices: the Simpson index (*D*) (Equation 2), Shannon-Weiner index (*H*) (Equation 3), Pielou index (Equation 4), and ln*S* index (Equation 5) (He et al., 2022; Sun et al., 2023). The calculation formulas are as follows:

(2) $$Simpson(D):\;D=1-\sum_{i=1}^{\text{n}}p_{i}^{2}$$

(3) $$\text{Shannon}-\text{Wiener}(H):\;H^{'}=-\sum_{i=1}^{S}P_{i}\ln P_{i}$$

(4) $$\text{Pielou}:\;J^{'}=H^{'}/\ln S$$

(5) lnS:           lnS

Where *S* is the total number of species in each quadrat, and *Pi* is the relative abundance of a species, with the subscript *i* ranging from 1 to *S*, representing each species in sequence.

### 2.4 Selection and measurement of functional traits

To quantify plant functional traits, we selected 63 herbaceous species representing six major community types during field surveys, adhering to standardized protocols (Pérez-Harguindeguy et al., 2016). We measured seven key traits pertaining to the leaf economics spectrum (**Supplementary Table 1**): leaf thickness (LT, mm), leaf area (LA, cm²), leaf length (LL, cm), leaf width (LW, cm), leaf carbon content (LC, g kg^-^¹), leaf nitrogen content (LN, g kg^-^¹), and leaf phosphorus content (LP, g kg^-^¹). Leaf thickness (LT) was measured at the leaf midpoint (between the lamina base and apex) using a digital caliper (accuracy: 0.01 mm). Three replicate measurements were taken and averaged for each sample (Cornelissen et al., 2003). Four to five fully expanded, healthy functional leaves free from pests and diseases were collected from each plant. Given the sparse distribution of certain plant species within desert steppe habitats, it proved difficult to guarantee the presence of ten plants per sample plot. Consequently, sampling was conducted based on the actual number of viable individuals within each plot, with the number of sampled plants per plot ranging from one to ten (Tayir et al., 2023). To minimize transpirational water loss, leaves were promptly sealed in plastic bags into which exhaled air was introduced to raise CO_2_ concentration and humidity. Later the same day, leaf area, leaf length, and leaf width were determined under humidified conditions using a calibrated portable leaf area meter (LI-3000C, Li-COR), with all raw data recorded. The dried material was ground to a fine powder using a ball mill. For the same species within a single plot, leaves collected from multiple healthy plants are thoroughly mixed to form a composite sample representing that species within the plot. Leaf carbon (LC) content was determined by the potassium dichromate heating method. Leaf nitrogen (LN) was quantified using the Kjeldahl method, and leaf phosphorus (LP) was assessed via perchloric acid digestion followed by molybdenum-antimony anti-colorimetric analysis (He et al., 2023b). The leaf C:N, C:P, and N:P ratios were subsequently calculated from these elemental concentrations. Plant functional traits were analyzed using the community-weighted mean (CWM) method. Based on the importance values of each species within individual plots (calculated by integrating relative height, relative density, relative cover, and relative biomass), weighted averages were computed for trait indices such as leaf length, leaf width, and leaf thickness. This involved multiplying the trait values of each species within a plot by its importance value proportion, then summing these products to obtain the community-weighted mean for each plot and trait. It should be noted that the data structure in this study is organized at the “species × plot” level: each data point in the figure represents the average trait value of a specific species within a given plot, weighted by its importance value, rather than being aggregated directly into a single plot-level value. This approach preserves trait variation information within the community, ensuring the integrity and transparency of the data structure (Violle et al., 2007).

### 2.5 Selection and measurement of soil

At each sampling point, we collected soil samples from the 0–10 cm layer using a soil sampler. After thorough homogenization and mixing into a single sample, 1000 grams were extracted for subsequent physicochemical analysis. We collected five replicate samples at each sampling point and measured soil bulk density *in situ* using the standard coring method. All samples were air-dried to constant weight indoors to determine soil organic carbon (SOC), total nitrogen (TN), and total phosphorus (TP) content. Dried samples were ground and sequentially sieved through 1 mm and 0.25 mm screens. Material passing the 0.25 mm sieve was used for all chemical analyses: SOC was determined using the potassium dichromate heating method (Zhang et al., 2021a). TN content was measured using the Kjeldahl method (Chen et al., 2022). TP content was analyzed via perchloric acid digestion followed by molybdenum-antimony anti-colorimetric determination (Zhang et al., 2022; Chen et al., 2025b). Soil water content (SWC) was calculated as the percentage weight loss after oven-drying at 105 °C to constant weight (Zheng et al., 2024).

### 2.6 Selection and measurement of climate

Climate data were obtained from the National Meteorological Science Data Center (http://www.cnern.org.cn). The dataset comprised daily records (1957–2022) from 35 reference stations across northern Xinjiang. To generate high-resolution, spatially continuous climate distribution maps, we employed a Geographically Weighted Regression (GWR) model for interpolation of key variables—annual mean temperature, annual precipitation, and annual mean wind speed. The model residuals were further corrected via inverse distance weighting (IDW). All resultant climate raster layers were subsequently adjusted for elevation effects. The robustness of the spatial interpolation was confirmed through model cross-validation. The procedure yielded coefficients of determination (*R²*) ranging from 0.76 to 0.88, demonstrating the high reliability and accuracy of the generated climate surfaces.

### 2.7 Statistical analysis

All primary calculations of plant diversity indices and functional traits were conducted using Microsoft Excel 2024 for data entry and preliminary processing. Statistical analyses were performed in SPSS 25.0, where one-way analysis of variance (ANOVA) with Turkey’s HSD *post-hoc* test (two-tailed) was used to compare species diversity indices across different months. Based on plot data, a linear mixed-effects model was constructed in R 4.3.3, with leaf traits as the response variable, elevation as a continuous covariate (i.e., a continuous predictor variable within the fixed effects), and month as a random effect. The *lmerTest* package was employed for model fitting and statistical inference. For multivariate analysis of environmental drivers, redundancy analysis (RDA) was performed using the *vegan* package, with significance of environmental variables evaluated through 999 permutation tests. Structural Equation Modeling (SEM) was performed using the *lavaan* package. Spatial interpolation and mapping of plant diversity and functional traits were conducted in Esri ArcGIS (version 10.8) using Kriging interpolation. Empirical Orthogonal Function (EOF) decomposition was applied to extract spatial patterns through eigenvalue decomposition, with the North criterion used to identify significant components. Additional analyses utilized several R packages: *dplyr* for data manipulation, *ggplot2* for data visualization, *ggpmisc* for statistical annotations, and *patchwork* for multi-panel figure arrangement.

## 3 Results

### 3.1 Response of plant diversity indices to seasonal dynamics

**Figure 2:**
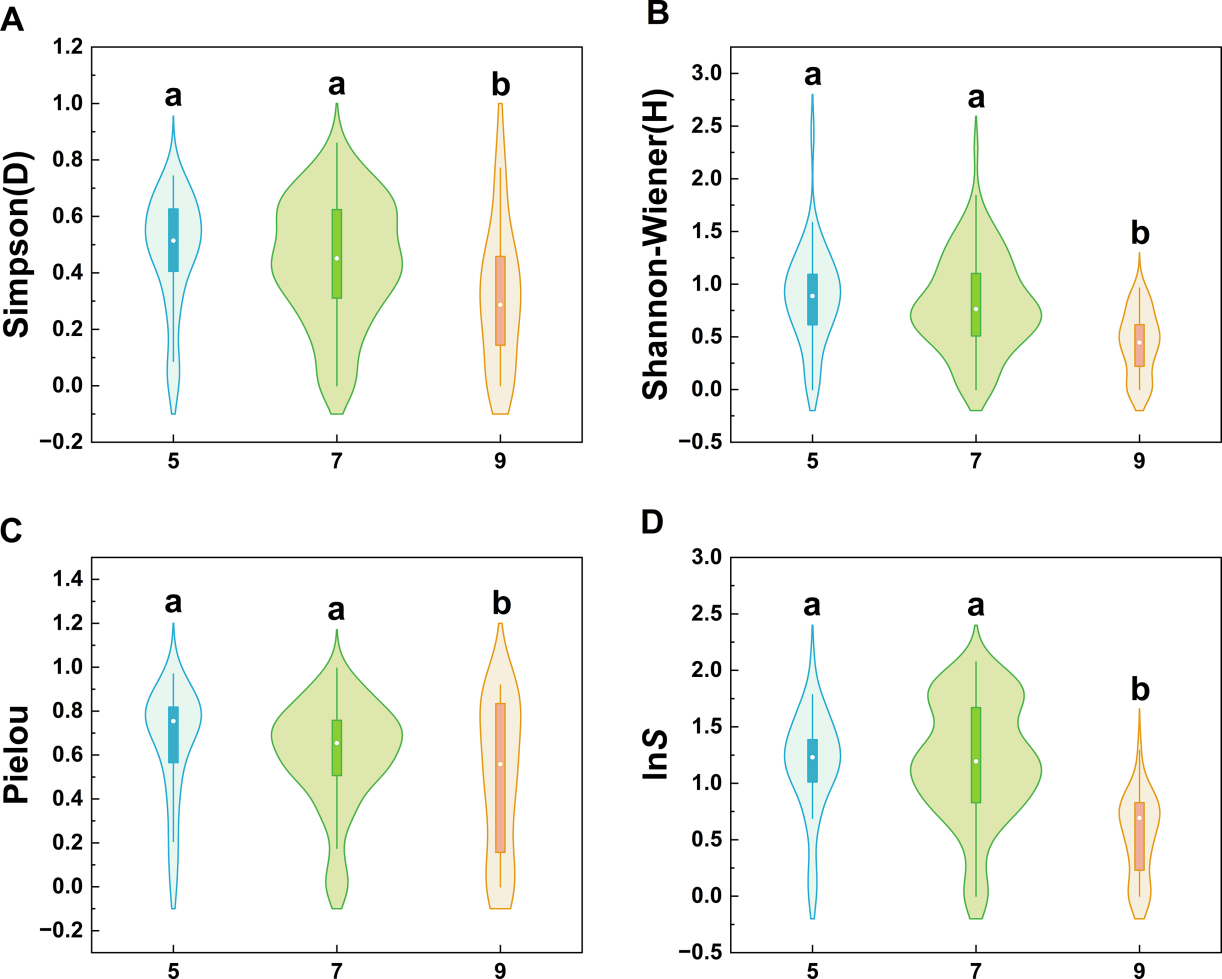
Community diversity index distribution across months. Violin plots of four diversity indices: **(A)** Simpson, **(B)** Shannon-Wiener, **(C)** Pielou, **(D)** ln*S*. X-axis: May, July, September (labeled 5, 7, 9); Y-axis: index values. Within each violin plot, the white dot denotes the median, the box represents the interquartile range (IQR, i.e. the 25th to 75th percentiles), and the whiskers extend to the minimum and maximum values (excluding outliers). Different lowercase letters (a, b) indicate significant monthly differences (*P* < 0.05, one-way ANOVA + Turkey’s test).

Plant diversity indices exhibited significant differences across sampling months (*P* < 0.05, **Figure 2**). Overall, diversity levels in May and July were comparable but both significantly higher than in September (**Supplementary Table 2**). Specifically, both the Simpson index(*D*) and Shannon-Wiener index(*H*) exhibited consistent seasonal decline trends: May and July values were significantly higher than September (*P* < 0.01), with *D* decreasing by 32.97% and 28.07%, respectively, and *H* decreasing by 50.24% and 46.04%, respectively. No significant differences were observed between May and July. Similarly, the ln*S* index in September decreased significantly by 54.28% and 55.67% compared to May and July, respectively (*P* < 0.01). The Pielou index also decreased by 25.48% (*P* < 0.01) and 18.96% (*P* < 0.05) compared to May and July, respectively. Furthermore, the ln*S* indices for all plant community types showed significant declines in September (**Supplementary Figure 2**).

### 3.2 Elevational patterns of leaf functional trait variation in desert steppe plant

**Figure 3:**
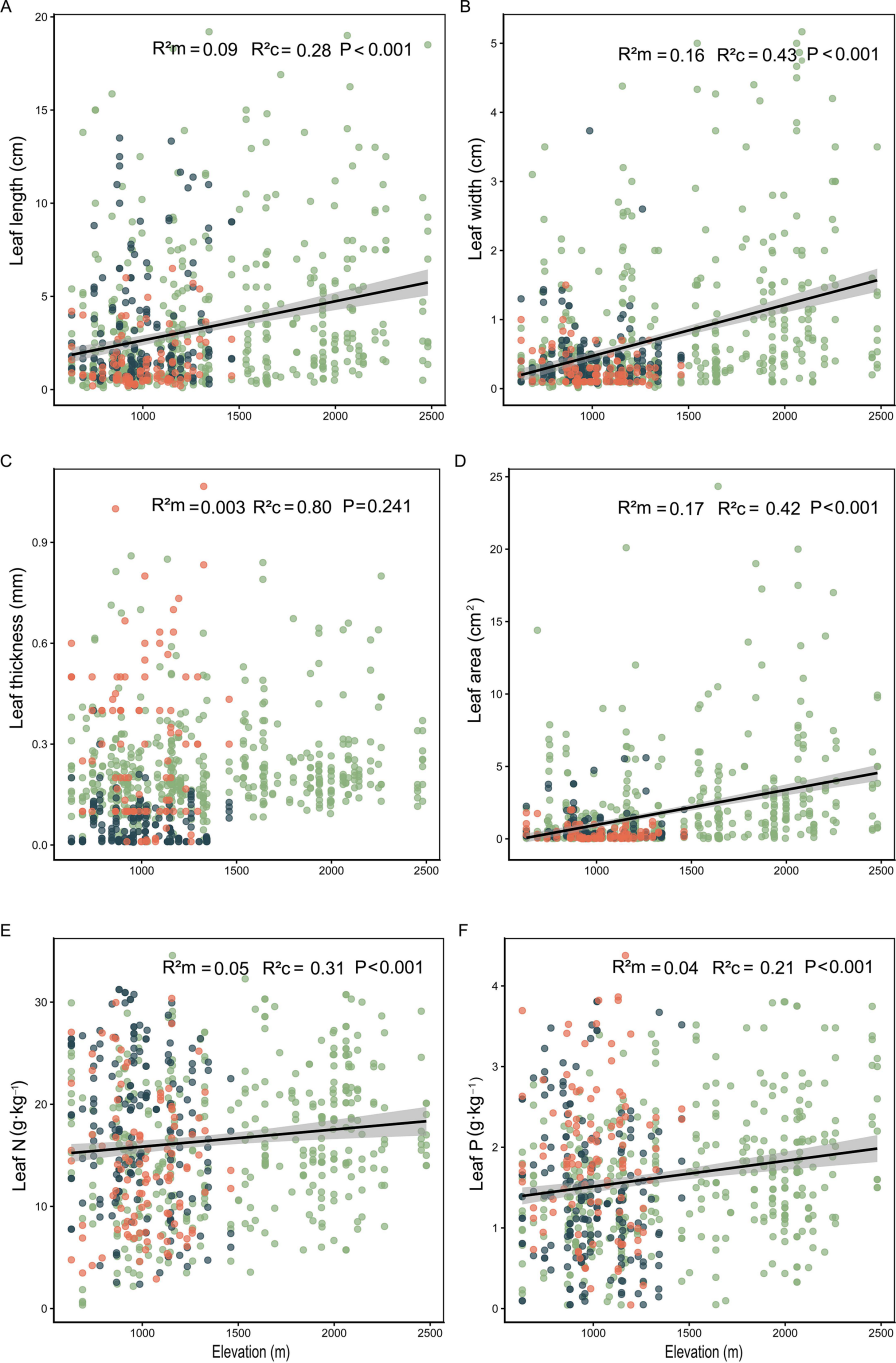
Elevational patterns of leaf functional trait variation in desert steppe plant. **(A)** Leaf length (cm); **(B)** Leaf width (cm); **(C)** Leaf thickness (mm); **(D)** Leaf area (cm^2^); **(E)** Leaf nitrogen content (g·kg^-1^); **(F)** Leaf phosphorus content (g·kg^-1^). Each data point represents the mean trait value for each species within a single plot, weighted by species importance value for analysis. Different colors denote distinct sampling months (blue dots indicate May, green dots indicate July, and orange dots indicate September). *R²m* (marginal *R*²) and *R²c* (conditional *R²*) were employed to assess model fit, with *P*-values indicating the significance level of regression relationships.

Overall, plant leaf functional traits exhibited a synergistic pattern of change along the elevational gradient, though variations were observed across different traits (**Figure 3**). Analysis of all sampled data revealed extremely significant positive correlations (P<0.001) between leaf length, leaf width, leaf area, leaf nitrogen content, and leaf phosphorus content with increasing elevation. Conversely, leaf thickness showed no significant correlation with elevation. Considering variations in sampling elevations across months (**Supplementary Figure 3**), we further examined monthly trends. Results indicate that in July, when the elevational gradient was most complete, all leaf functional traits (including leaf length, width, thickness, area, nitrogen content, and phosphorus content) showed extremely significant positive correlations with elevation (P<0.005). Conversely, in May and September, where the elevational range was relatively narrow, no significant correlations between traits and elevation were detected.

### 3.3 Spatial patterns of plant diversity and functional traits

**Figure 4:**
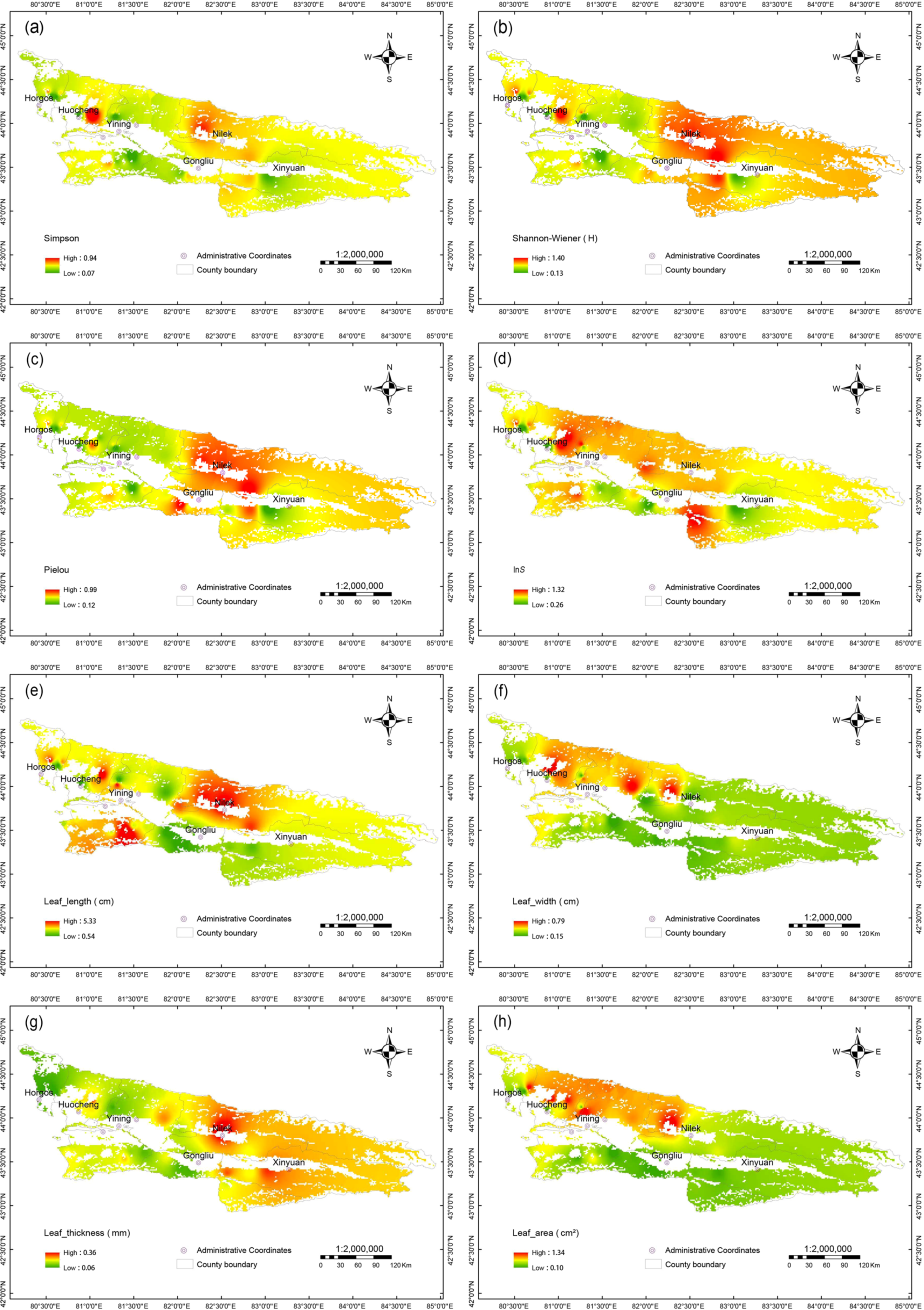
Spatial distribution of plant functional traits and diversity indices in the desert steppe of the Ili River basin. **(A)** Spatial pattern of Simpson index(*D*); **(B)** Spatial pattern of Shannon-Wiener index(*H*); **(C)** Spatial pattern of Pielou index; **(D)** Spatial pattern of ln*S* index; **(E)** Spatial pattern of leaf length (cm); **(F)** Spatial pattern of leaf width (cm); **(G)** Spatial pattern of leaf thickness (mm); **(H)** Spatial pattern of leaf area (cm²). The color gradient from green to red represents the range from low to high values of each index. Administrative Coordinates indicate the locations of counties (Horgos, Huocheng, Yining, Nilek, Gongliu, Xinyuan), and County Boundary represents the county administrative boundaries. The scale is 1:2,000,000.

We found that plant diversity indices (Simpson(*D*), Shannon–Wiener(*H*), Pielou, and ln*S*) and spatial heterogeneity of leaf functional traits (leaf length, width, thickness, and area) exhibited dynamic characteristics (**Figure 4**). Overall, both plant diversity and functional traits exhibited patchy mosaic patterns with significant spatial heterogeneity. High values of plant functional traits were mainly concentrated in Nilek (a–d), while low values (except for leaf thickness, which reached its minimum of 0.06 mm in Horgos) were mainly distributed in Gongliu and Xinyuan. Similarly, high values of plant diversity were also primarily found in Nilek, distributed in a gradient extending toward southeastern Nilek County and the surrounding areas of Gongliu, whereas low values were scattered, mainly in Xinyuan and Yining. In terms of seasonal dynamics, July was the period with the most prominent spatial heterogeneity. Plant diversity showed core high-value areas in the central-southern region (**Supplementary Figure 4**). Notably, in July, most areas of the study region exhibited low ln*S* values except around Huocheng. By September, nearly the entire survey area showed low values. For plant functional traits, low values in July were concentrated in Gongliu (**Supplementary Figure 5**). High values of leaf length, width, and area were clustered around Yining and Nilek, while relatively higher values for leaf thickness were mainly distributed in Xinyuan.

### 3.4 Response of plant diversity and functional traits to environmental factors

**Figure 5:**
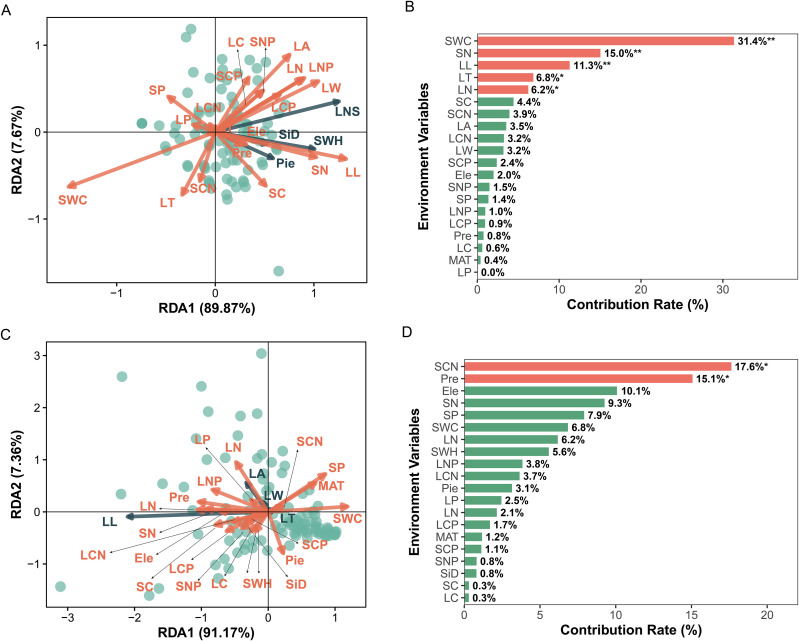
Redundancy analysis (RDA) and contribution of environmental variables to plant diversity and plant functional traits **(A, C)**. Redundancy analysis (RDA) ordination diagrams depicting the associations between plant functional traits (dots) and environmental variables (arrows). RDA1 and RDA2 represent the first and second canonical axes, with their explained variance percentages in parentheses. **(B, D)** Contribution rate bar graphs of environmental variables to the total variance of plant functional traits. Significance of contributions is indicated by asterisks: *P < 0.05, **P < 0.01.

In the RDA analysis of plant diversity, RDA1 and RDA2 explained 89.87% and 7.67% of the total variance, respectively, with RDA1 serving as the primary explanatory axis (**Figure 5A**). Soil nitrogen content showed a highly significant positive correlation with leaf nitrogen content, with both variables pointing in the same direction on the RDA1 axis, indicating that increased soil nitrogen significantly elevated leaf nitrogen content. Soil water content (SWC) showed significant positive correlations with both leaf length (LL) and leaf thickness (LT), with all three variables covarying along RDA1. This indicated that increased soil moisture substantially promotes growth in leaf length and thickness. Further variance decomposition analysis (**Figure 5B**) revealed that SWC (31.40%), soil carbon (SC, 15.00%), and LL (11.30%) were highly significant factors (*P* < 0.01), while LT (6.80%) and leaf nitrogen (LN, 6.20%) were significant factors (*P* < 0.05).

In the RDA analysis of plant functional traits, RDA1 and RDA2 explained 91.17% and 7.36% of the total variance, respectively (**Figure 5C**), with RDA1 demonstrating stronger explanatory power as the core axis. Soil carbon-to-nitrogen ratio (SCN) showed a significant positive correlation with precipitation (Pre), with both variables pointing in the same direction on RDA1. This indicates that increased precipitation significantly elevated the soil carbon-to-nitrogen ratio. Variation decomposition analysis (**Figure 5D**) identified SCN (17.60%) and Pre (15.10%) as significant influencing factors (*P* < 0.05). Additionally, elevation (Ele, 10.10%), soil nitrogen (SN, 9.30%), soil phosphorus (SP, 7.90%), and SWC (6.80%) also contributed significantly to explained variation. In summary, environmental variables exhibited significant differences in their explanatory contributions to plant diversity and functional traits. Soil water content, soil chemical nutrients, and precipitation factors consistently demonstrated high and statistically significant contributions across both analyses, confirming them as key environmental drivers of plant diversity and functional trait variation.

In the figures, the plant diversity indices are abbreviated as follows: Simpson index (*D*): SID; Shannon-Wiener index (*H*): SWH; Pielou index: Pie; ln*S* index: LNS. Abbreviations for plant functional traits include leaf length (LL), leaf width (LW), leaf thickness (LT), leaf area (LA), leaf carbon content (LC), leaf nitrogen content (LN), leaf phosphorus content (LP), leaf C:N ratio (LCN), leaf N:P ratio (LNP) and leaf C:P ratio (LCP). Soil properties are abbreviated as follows: soil water content (SWC); soil carbon content (SC); soil nitrogen content (SN); soil phosphorus content (SP); soil C:N ratio (SCN); soil N:P ratio (SNP); and soil C:P ratio (SCP). Climate and topography variables are: precipitation (Pre), mean annual temperature (MAT) and elevation (Ele).

**Figure 6:**
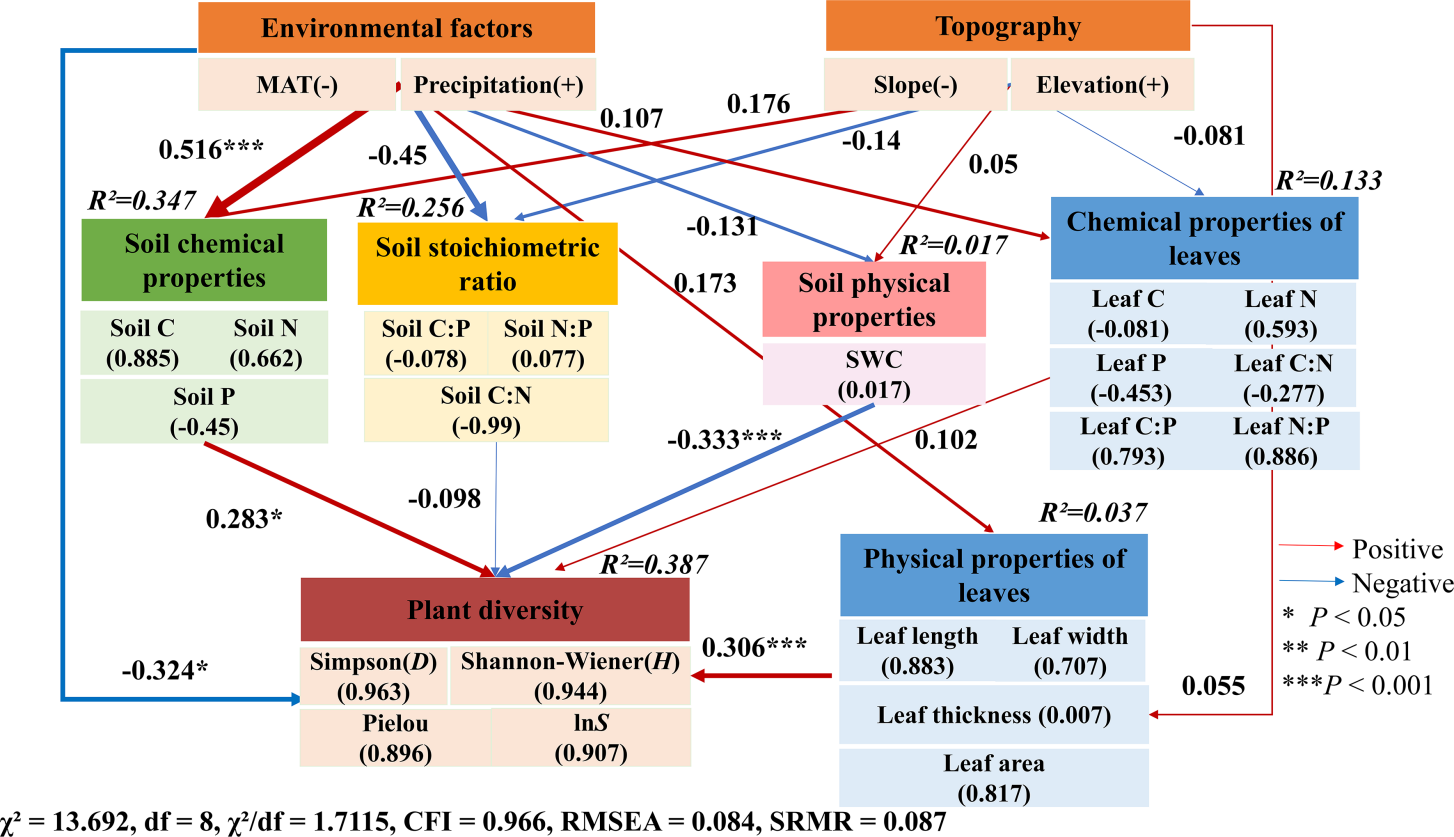
Structural Equation Model (SEM) showing the effects of climate, soil properties with on biological diversity and functional traits. *R^2^* values represent the proportion of variance explained for each dependent variable in the model. Numbers indicate the standard path coefficient of the pathways, with arrow width proportional to the strength of the relationship. Red arrows represent positive effects, blue arrows represent negative effects. Significance levels are indicated by *, **, and *** for *P* < 0.05, *P* < 0.01 and *P* < 0.001, respectively.

Structural Equation Modeling (SEM) indicated that variations in plant diversity were primarily driven by climatic factors via their influence on soil properties, which, together with functional traits, collectively accounted for 38.7% of the total variance (**Figure 6**). Notably, both climate and soil factors exerted direct regulatory effects on plant diversity. Additionally, changes in plant morpho-functional traits were predominantly driven by precipitation, explaining 30.7% of the observed variation (*χ^2^* = 13.692, df=8, CFI = 0.988, RMSEA = 0.084, *P* = 0.0901).

## 4 Discussion

### 4.1 Response of plant diversity in different community types to seasonal dynamics

We have discovered that environmental selection (particularly seasonal variations in water availability) is a core driver shaping patterns of plant diversity, constituting the fundamental mechanism underpinning adaptive strategies in plant community formation (Currier and Sala, 2022). Based on this, we interpret the results from two perspectives: the community as a whole and the dominant family community types, systematically investigating the integrated ecological strategies employed by plants in response to seasonal stress. At the community level, plant diversity exhibited distinct seasonal patterns that correspond to the dynamic hydrothermal conditions. Diversity remained relatively high in May and July but decreased significantly in September (**Figure 2**), closely aligning with seasonal humidity and temperature fluctuations. This strongly indicates that the onset of the dry season (September) imposes intense environmental selection through water stress, a pattern consistent with findings from multiple grassland ecosystems globally where seasonal precipitation is the primary driver (Ogle and Reynolds, 2004; Xu and Baldocchi, 2004). This study further reveals the differences in response strategies among different dominant community types (**Supplementary Figure 1**). Communities dominated by Asteraceae maintained their dominant status throughout all sampling months. Its key species, *Seriphidium transiliense*, displayed highly drought-tolerant traits, indicating directional environmental selection for specific functional characteristics. In contrast, plant communities dominated by Poaceae, Cyperaceae, and Chenopodiaceae exhibit relatively stable seasonal dynamics (**Supplementary Figure 2**). Species within communities dominated by Poaceae and Cyperaceae maintain population stability through clonal growth strategies, wherein root bud primordia differentiate into adventitious roots, subsequently producing genetically identical tillers (Terer et al., 2015; Arnillas et al., 2021; Su et al., 2022; Wang et al., 2025a). In communities dominated by Chenopodiaceae, the primary plants employ unique physiological and morphological adaptation mechanisms. These include selectively absorbing ions through root cell membrane channels (prioritizing potassium uptake while restricting harmful ions), and utilizing specialized structures to reduce salt intrusion and water loss. This alleviates osmotic stress, enabling ecological niche occupation (Katembe et al., 1998; Dehghanian et al., 2024). These differential strategies ultimately drive seasonal shifts in community dominance. Our findings align with reports from Yanchi, China (Zhou et al., 2015), Inner Mongolia, China (Gao et al., 2024), and Iran (Mahdavi et al., 2013), further confirming that dominant species secure broader ecological niches through specific adaptive traits (García Criado et al., 2025). Under similar arid and semi-arid climatic conditions, environmental filtering tends to select dominant families with different strategies, enabling them to occupy a dominant position within their respective communities. Collectively, this study emphasizes that comprehensive seasonal monitoring is critical for accurately understanding how plant diversity responds to environmental changes in desert steppe ecosystems.

### 4.2 Dynamic trade-offs in plant functional traits along elevation under seasonal variability

Our findings demonstrate a significant positive relationship between key leaf functional traits (Leaf length, width, area, nitrogen content, phosphorus content) and elevation in the desert steppe (**Figure 3**), pointing to adaptations for survival in variable habitats. This trend reflects a widespread ecological strategy employed by plants in response to environmental stress (Poorter et al., 2008; Bloomfield et al., 2018; Sigdel et al., 2023), and is supported by recent studies (Rixen et al., 2022; Liu et al., 2023), collectively confirming the pivotal role of adaptive adjustments in leaf traits for improving plant adaptation to heterogeneous environments (Jiang et al., 2021). Moving beyond this static pattern, our study uncovers pronounced seasonal variation in trait-elevation relationships, offering deeper mechanistic insight. The absence of a clear elevational pattern reported by Xu et al (Xu et al., 2018). in the Taibai Mountains contrasts with our findings. We attribute this discrepancy to differences in ecosystem composition and habitat heterogeneity. More fundamentally, it underscores that single-time-point sampling can obscure the full spectrum of trait responses. Our multi-temporal observations captured these dynamic adjustments across the growing season (**Supplementary Figure 3**).

Overall, plant functional traits exhibit a pronounced upward trend along the elevational gradient, seemingly at odds with the leaf economic spectrum (LES) prediction that higher elevations favor resource-conservative strategies. To explain this phenomenon, we investigated the seasonal dynamics of trait-elevation relationships and identified significant inter-monthly variations. This seasonal heterogeneity primarily stems from the unique hydrothermal conditions during the peak growth period in July: warming temperatures, increased precipitation, and high-elevation snowmelt collectively enhance soil water availability, temporarily alleviating the water stress prevalent in desert grasslands. Within this window of reduced water constraints, plants tend to enhance the morphology of photosynthetic organs and increase leaf nutrient content. This maximizes photosynthetic efficiency within the limited growing season, ensuring the completion of their life cycle (Díaz et al., 2016). Specifically, leaf length, width, area, and nitrogen-phosphorus content all increase significantly with elevation (**Supplementary Figure 3**), reflecting a typical rapid resource acquisition strategy (**Supplementary Figure 9**). This correlates closely with the prevalence of annual plants in desert grasslands. Such species must rapidly complete vegetative growth during July’s optimal moisture window to accumulate resources for subsequent reproduction. This finding supports our hypothesis: during the environmentally optimal peak growth period, high-elevation plants temporarily deviate from conservative strategies, adopting instead a maximized photosynthetic investment approach to complete their life cycles. In contrast, the correlation between traits and elevation significantly weakened or even disappeared in May and September, primarily regulated by phenological processes. In May, although plants break dormancy, low temperatures constrain full leaf expansion and physiological recovery, leading to unstable trait expression and preventing the formation of an elevational gradient (Qiu et al., 2024). By September, the desert steppe enters the dry season with sharply reduced precipitation, re-establishing water stress as the dominant factor. Plants begin shifting from vegetative growth towards reproductive allocation or dormancy induction (Khatri et al., 2022), with resource allocation reverting to the conservative traits described by the leaf economics spectrum.

To further validate the aforementioned inference, we conducted differential analyses of leaf functional traits across months (**Supplementary Figure 6**). Results indicated that leaf length, width, and area in July were significantly higher than in May and September, corroborating enhanced photosynthetic investment during the growth peak. Conversely, leaf thickness and phosphorus content in September were markedly higher than in other months, aligning with the pattern of plants adopting conservative strategies to bolster stress resistance under adverse conditions (Cui et al., 2020). Collectively, these findings support the hypothesis that plant strategies dynamically shift in response to environmental conditions and phenological stages. In summary, elevational adaptation in desert steppe plants does not follow a singular resource allocation pattern. Instead, driven by environmental and phenological factors, it involves a dynamic trade-off between rapid acquisition and conservative usage. By tracing seasonal variations, this study clearly reveals the complete process of strategic transition in plants, from early growth stages through peak growth to seasonal decline.

### 4.3 Spatial patterns and influencing factors of plant diversity and functional traits

This study reveals a significant spatial synergy between plant diversity and functional traits in the desert steppe, characterized by a distinct patchy mosaic distribution (**Figure 4**). This coordinated heterogeneity underscores the multi-dimensional role of environmental filtering in shaping community assembly. As fundamental indicators of ecosystem functioning, the spatially coupled relationship between diversity and traits jointly reflects ecosystem stability, a concept supported by prior research (Mayfield et al., 2010; Ren et al., 2015). Geographically, the highest values for both parameters clustered in Nilek County, while the lowest values were concentrated in Xinyuan and Gongliu Counties. The relatively favorable hydrothermal conditions in Nilek County are known to foster plant resource-acquisition strategies. Consistent with this, our study found a concurrent enhancement of diversity and trait expression there, with representative species such as *Taraxacum mongolicum* and *Chorispora tenell* exhibiting markedly larger leaf traits. In contrast, intense spring and autumn grazing pressure in Xinyuan and Gongliu Counties suppresses plant growth rates (Zuo et al., 2018), which in our study was associated with limited establishment of additional species. The desert steppe serves as a unique “spring-autumn pasture” where ecological productivity is primarily driven by the “pulse growth” of ephemeral plants. This phenomenon is highly dependent on spring snowmelt and exhibits significant spatial heterogeneity. When coupled with high evaporation and low precipitation conversion efficiency, these conditions drive plants toward smaller leaves to minimize water loss (Bjorkman et al., 2018; Yan et al., 2023), ultimately reducing community diversity (Laliberté et al., 2014; Haghpanah et al., 2024). The observed decline in species richness (ln*S*) at both the overall community (**Supplementary Figure 4****(l)**) and functional group levels (**Supplementary Figure 2**) confirms intensified interspecific competition under stress and the consequent dominance of a few stress-tolerant species (Zhou et al., 2025). Consequently, plant diversity and functional traits are interdependent. Their adaptive synergy, which is achieved through coordinated variation, ultimately underpins the core mechanism by which plants respond to environmental heterogeneity. Therefore, these spatial maps are not only records of ecological phenomena but also a basis for defining functional zones. They provide practical guidance for government sectors to identify “fragile ecological areas” and “efficient restoration zones”.

Redundancy analysis (RDA) elucidated the multi-scale environmental drivers (**Figure 5**). At the local scale, soil water content (SWC) emerged as the primary factor, showing significant positive correlations with multiple functional traits. This aligns with the established consensus that soil moisture is a critical regulator of plant growth in arid regions (Sala et al., 1988; Liu et al., 2025). Crucially, our finding that SWC concurrently shaped both community-level traits and diversity extends this understanding and provides direct empirical support for the integrative hypothesis proposed by Zeppel et al (Zeppel et al., 2014). At the regional scale, the influences of the soil carbon-to-nitrogen ratio (SCN), precipitation (Pre), and elevation (Ele) became predominant, highlighting the dominant role of macro-climatic and topographic factors in governing large-scale patterns. This confirms that water and nutrient availability are pivotal in directing plant adaptive strategies. By steering traits toward conservative strategies and reducing diversity, these environmental filters may thereby compromise ecosystem functioning and stability, as suggested by recent studies (Xu et al., 2014; Luo et al., 2021). Concurrently, the plant diversity index exhibited significant correlations with both leaf length and width, as well as leaf nitrogen content, further underscoring the inextricable link between plant diversity and functional traits (**Figure 5B**).

Furthermore, the SEM results proved that the soil nutrient was mainly mediated the plant diversity in the desert steppes (**Figure 5**). In desert steppes, the environmental drivers of plant communities exhibit a clear hierarchical scale effect. At the local scale, micro-topography governs the redistribution of soil water content (SWC), directly modulating the immediate physiological status and functional performance of plants. However, at the regional scale, following precipitation gradients, the soil nutrient cycling rate (e.g., SCN) emerges as the “ultimate filter” for species distribution and biodiversity assembly (Wu et al., 2019; Guo et al., 2024). This suggests that while water availability determines the survival threshold, nutrient supply dictates the upper limit of community diversity. Our Structural Equation Model (SEM) further confirms that climatic factors do not always act directly on individual plants but instead exert influence through below-ground mediation (**Figure 6**). Fluctuations in precipitation and temperature alter soil weathering, nutrient mineralization, and microbial activity, which in turn reconfigure the plant-available resource pool (Wang et al., 2024). This mediating role of soil nutrients provides robust evidence for the profound control of subsurface biogeochemical processes over above-ground biodiversity in arid ecosystems. Under the stress of reduced precipitation or nutrient scarcity, plants exhibit a clear trade-off strategy. By reducing leaf dimensions (length and width) and increasing nutrient investment (Li et al., 2020), plants transition from an “acquisitive” to a “conservative” strategy. This priority of functional plasticity over taxonomic turnover suggests that morphological adjustment is the primary adaptive response to environmental fluctuations (Guo et al., 2022; Henn et al., 2018; Wilcox et al., 2021). While these conservative strategies ensure species persistence, they often come at the expense of total community productivity, potentially leading to a decline in overall ecosystem functioning.

In summary, this study deciphers the synergistic spatial patterns between plant diversity and functional traits, revealing a hierarchical structure of environmental drivers across different scales. Our findings highlight that while localized water availability dictates immediate functional responses, regional climatic and edaphic filters govern the broader assembly of biodiversity. By elucidating these causal pathways, this research provides a robust theoretical foundation for developing spatially differentiated management and targeted conservation strategies for desert steppe ecosystems in the face of global change.

## 5 Conclusion and future directions

This study reveals that the adaptation of desert steppe ecosystems to environmental heterogeneity is a dynamic process, centered on pronounced spatio-temporal divergence in plant diversity and functional traits. Specifically, plant diversity exhibits distinct seasonal dynamics: it declines markedly during the dry season, but increases during periods of favorable hydrothermal conditions, with significantly higher species richness in the latter. Concurrently, plant functional traits show an overall upward trend along the elevational gradient, a pattern similarly modulated by seasonal variations. Based on the leaf economics spectrum, further analysis indicates that leaf morphological traits play a crucial regulatory role in maintaining diversity, underscoring the intrinsic link between functional attributes and community composition. Spatially, the observed patchy mosaic structure is primarily driven by the synergistic effects of soil moisture, nutrient balance, and climatic factors. Based on these findings, we recommend that future research combine underground bud bank surveys with multi-year observations to more comprehensively reveal the dynamics of species change. At the same time, we emphasize the need to shift ecological monitoring from static surveys towards dynamic tracking of key traits and community types. When combined with spatially differentiated management, this approach will facilitate the formulation of precise conservation strategies, thereby promoting the sustainable restoration and development of globally vulnerable grasslands.

## Data Availability

The raw data supporting the conclusions of this article will be made available by the authors, without undue reservation.
