# Supplementary material for: Spatiotemporal dynamics and adaptive strategies of plant diversity and functional traits in desert steppe
Source: Front Plant Sci. 2026 Apr 17;17:1763260. doi: 10.3389/fpls.2026.1763260 (PMC13133033; doi:10.3389/fpls.2026.1763260)
Supplement: Supplementary file 1 [file DataSheet1.docx]

***Supplementary Material***

**SUPPLEMENTARY TABLES AND FIGURES**

**Composition of plant family**

An analysis of desert steppe plants in the Ili River basin has categorised them into six major plant families ([Table S1](#TableS1)): Asteraceae dominated by *Seriphidium transiliens*, Chenopodiaceae dominated by *Ceratocarpus arenarius*, Poaceae dominated by *Stipa capillata*, Cyperaceae represented solely by *Carex liparocarpos*, Fabaceae dominated by *Sophora alopecuroides,* and Others dominated by *Peganum harmala* .

**Supplementary Table 1**

**Table S1.** **Composition of Plant families in Desert Steppe.** This table lists the plant species composition across different plant families. For each families, constituent species and high-frequency dominant species are identified. This table lists the species composition of different plant families (Asteraceae, Chenopodiaceae, Poaceae, Cyperaceae, Fabaceae, and Others). For each plant family, both constituent species and high-frequency dominant species are listed.

| Plant family classification | Plant family composition | High-frequency dominant species |
| --- | --- | --- |
| Asteraceae | *Seriphidium transiliense, Taraxacum mongolicum, Artemisia frigida, Achillea millefolium, Filago arvensis, Artemisia scoparia, Chondrilla piptocoma, Artemisia gmelinii , Cirsium spicatum, Aster hispidus, Symphyotrichum ciliatum, Convolvulus arvensis* | *Seriphidium transiliense* |
| Chenopodiaceae | *Ceratocarpus arenarius, Krascheninnikovia ceratoides, Salsola tragus, Petrosimonia sibirica, Grubovia dasyphylla, Bassia prostrata, Oxybasis glauca* | *Ceratocarpus arenarius* |
| Poaceae | *Elymus nutans, Bromus inermis, Neotrinia splendens, Festuca ovina, Stipa capillata, Poa annua, Phleum pratense, Cynodon dactylon, Aegilops tauschii, Chloris virgata, Eremopyrum triticeum* | *Stipa capillata* |
| Cyperaceae | *Carex liparocarpos* | *Carex liparocarpos* |
| Fabaceae | *Trigonella arcuata, Sophora alopecuroides, Medicago sativa, Medicago falcata, Astragalus membranaceus, Alhagi camelorum* | *Sophora alopecuroides* |
| Others | *Myosotis alpestris, Galium spurium , Arenaria serpyllifolia, Strigosella africana, Androsace umbellata,Chorispora tenella, Phlomoides umbrosa, Alchemilla japonica, Rubia cordifolia, Anethum graveolens, Polygonum aviculare, Tribulus terrestris, Halogeton arachnoideus, Plantago asiatica, Peganum harmala, Lappula myosotis, Euphrasia pectinata, Peganum harmala, Geranium wilfordii, Veronica campylopoda, Iris tectorum, Cerastium arvense, Thalictrum aquilegiifolium, Fragaria vesca, Sibbaldianthe bifurca, Bassia prostrata.* | *Peganum harmala* |

**Temporal patterns of plant diversity across months.**

Plant diversity indices exhibited significant variations across the sampling months, revealing distinct seasonal dynamics ([Table S2](#TableS2)). Overall, a marked decline in plant diversity was observed in September. Specifically, the Simpson (*D*) index showed a significant decreasing trend over time, with values in both May and July being significantly higher than those in September, decreasing by 32.97% (*P* < 0.01) and 28.07% (*P* < 0.01), respectively, while no significant difference was detected between July and May. Similarly, the Shannon–Wiener index (*H*) was significantly higher in May and July than in September, with reductions of 50.24% (*P* < 0.01) and 46.04% (*P* < 0.01), respectively. And ln*S* index showed a pronounced reduction in September, dropping by 54.28% (*P* < 0.01) and 55.67% (*P* < 0.01) relative to May and July, respectively. In addition, the Pielou index also decreased significantly in September compared to May and July, with declines of 25.48% (*P* < 0.01) and 18.96% (*P* < 0.05), respectively.

**Supplementary Table 2**

**Table S2. Monthly variation in plant diversity.** Mean ± standard error of species diversity index for different months. Different lowercase letters denote significant differences between months (*P* < 0.05)

| Month | Simpson(*D*) | Shannon-Wiener(*H*) | Pielou | ln*S* |
| --- | --- | --- | --- | --- |
| May | 0.477 ± 0.030^a^ | 0.874 ± 0.069^a^ | 0.654 ± 0.038^a^ | 1.162 ± 0.068^a^ |
| Jul. | 0.444± 0.021^a^ | 0.806 ± 0.045^a^ | 0.601 ± 0.023^a^ | 1.199 ± 0.051^a^ |
| Sep. | 0.320 ± 0.045^b^ | 0.435 ± 0.053^b^ | 0.487 ± 0.060^b^ | 0.531 ± 0.063^b^ |

**Supplementary Table 3**

**Table S3. Morphological and chemical traits of leaves in different plant species.** Data in the table are presented as “mean ± standard deviation.” Leaf traits include leaf length, leaf width, leaf thickness, leaf area, leaf nitrogen content, and leaf phosphorus content.

| Species | Leaf length | Leaf width | Leaf thickness | Leaf area | Leaf Nitrogen | Leaf phosphorus |
| --- | --- | --- | --- | --- | --- | --- |
| *Seriphidium transiliense* | 0.99±0.68 | 0.25±0.24 | 0.13±0.19 | 0.12±0.10 | 15.73±6.10 | 1.94±1.60 |
| *Artemisia frigida* | 0.95±NA | 0.10±NA | 0.08±NA | 0.8±NA | 24.05±NA | 1.11±NA |
| *Salsola tragus* | 0.92±0.55 | 0.11±0.04 | 0.10±0.08 | 0.12±0.12 | 8.87±4.79 | 0.85±0.05 |
| *Myosotis alpestris* | 3.23±3.81 | 0.42±0.19 | 0.05±0.04 | 0.49±0.51 | 12.82±6.47 | 1.14±0.67 |
| *Petrosimonia sibirica* | 1.97±0.55 | 0.30±0.15 | 0.07±0.07 | 0.25±0.12 | 20.93±7.94 | 1.75±1.61 |
| *Elymus nutans* | 5.81±3.47 | 0.41±0.23 | 0.06±0.05 | 1.22±1.22 | 16.22±7.33 | 1.47±1.15 |
| *Cirsium spicatum* | 5.74±1.77 | 2.42±0.58 | 0.67±0.22 | 8.04±5.14 | 15.57±13.67 | 7.41±7.81 |
| *Achillea millefolium* | 1.96±1.38 | 0.61±0.53 | 0.26±0.13 | 0.88±0.79 | 23.31±8.81 | 1.72±1.35 |
| *Trigonella arcuata* | 0.64±0.27 | 0.44±0.10 | 0.05±0.04 | 0.38±0.31 | 21.55±8.19 | 2.12±1.62 |
| *Galium spurium* | 3.45±0.05 | 0.83±0.10 | 0.12±0.03 | 1.57±0.05 | 12.90±1.03 | 1.12±0.05 |
| *Arenaria serpyllifolia* | 0.26±0.09 | 0.12±0.03 | 0.01±0.00 | 0.08±0.04 | 20.56±5.88 | 0.89±0.70 |
| *Bromus inermis* | 2.32±1.03 | 0.15±0.05 | 0.06±0.04 | 0.34±0.32 | 10.89±4.41 | 0.92±0.28 |
| *Poa annua* | 3.77±1.22 | 0.44±0.15 | 0.09±0.04 | 1.13±0.34 | 9.04±3.21 | 2.71±1.31 |
| *Eremopyrum triticeum* | 5.37±3.21 | 0.43±0.29 | 0.12±0.19 | 1.13±1.50 | 14.32±7.54 | 1.70±1.01 |
| *Bassia prostrata* | 0.88±0.49 | 0.12±0.06 | 0.20±0.09 | 0.14±0.09 | 14.60±7.65 | 3.00±4.74 |
| *Phleum pratense* | 3.51±1.20 | 0.41±0.17 | 0.10±0.05 | 0.88±0.78 | 12.08±6.48 | 1.68±0.80 |
| *Strigosella africana* | 1.60±NA | 0.45±NA | 0.31±NA | 0.32±NA | 18.10±NA | 1.62±NA |
| *Oxybasis glauca* | 1.73±0.64 | 0.72±0.13 | 0.25±0.25 | 1.27±0.88 | 20.52±9.09 | 1.12±0.45 |
| *Androsace umbellata* | 1.07±0.60 | 0.11±0.026 | 0.12±0.06 | 0.12±0.08 | 17.31±3.72 | 1.59±0.37 |
| *Aster hispidus* | 1.52±0.38 | 0.38±0.19 | 0.31±0.16 | 0.52±0.26 | 26.26±3.98 | 2.64±0.54 |
| *Cynodon dactylon* | 5.38±3.01 | 0.32±0.05 | 0.21±0.12 | 1.42±0.18 | 3.28±NA | 0.74±NA |
| *Artemisia scoparia* | 1.07±0.10 | 0.15±0.10 | 0.13±0.04 | 0.08±0.03 | 21.34±6.07 | 1.13±0.26 |
| *Convolvulus arvensis* | 2.12±NA | 0.38±NA | 0.28±NA | 1.25±NA | 9.90±NA | 0.38±NA |
| *Artemisia gmelinii* | 3.80±NA | 1.25±NA | 0.12±NA | 0.34±NA | 17.88±NA | 3.22±NA |
| *Symphyotrichum ciliatum* | 1.32±0.41 | 0.24±0.08 | 0.09±0.09 | 0.17±0.10 | 18.51±7.42 | 1.83±0.88 |
| *Chorispora tenella* | 1.74±0.98 | 0.45±0.16 | 0.07±0.06 | 0.70±0.57 | 22.61±3.73 | 1.33±1.01 |
| *Chondrilla piptocoma* | 1.99±NA | 0.25±NA | 0.14±NA | 0.14±NA | 0.34±NA | 2.77±NA |
| *Phlomoides umbrosa* | 5.75±3.18 | 2.15±0.78 | 0.60±0.35 | 6.13±4.07 | 18.18±14.60 | 1.48±0.66 |
| *Filago arvensis* | 0.78±1.01 | 0.31±0.52 | 0.19±0.14 | 0.57±1.74 | 7.60±5.00 | 1.22±0.71 |
| *Festuca ovina* | 3.88±1.76 | 0.14±0.09 | 0.13±0.06 | 0.42±0.42 | 8.79±3.71 | 1.18±0.82 |
| *Alchemilla japonica* | 3.01±1.42 | 4.92±2.75 | 0.33±0.10 | 13.80±14.14 | 18.74±4.30 | 2.07±1.27 |
| *Aegilops tauschii* | 3.28±1.03 | 0.23±0.11 | 0.17±0.07 | 0.63±0.42 | 9.21±5.46 | 2.40±3.49 |
| *Neotrinia splendens* | 22.24±9.81 | 0.37±0.07 | 0.61±0.51 | 6.85±4.31 | 13.03±5.83 | 1.19±1.02 |
| *Medicago sativa* | 1.53±1.28 | 0.41±0.18 | 0.15±0.15 | 0.45±0.27 | 25.05±13.46 | 1.19±0.69 |
| *Sophora alopecuroides* | 1.93±0.57 | 0.86±0.30 | 0.26±0.17 | 1.62±1.30 | 24.38±5.33 | 1.53±0.80 |
| *Rubia cordifolia* | 1.00±0.42 | 0.19±0.12 | 0.08±0.10 | 0.12±0.11 | 15.78±2.28 | 1.32±0.18 |
| *Carex liparocarpos* | 6.34±3.49 | 0.26±0.13 | 0.19±0.18 | 1.05±1.08 | 14.34±6.59 | 1.76±2.04 |
| *Anethum graveolens* | 3.65±0.05 | 0.07±0.03 | 0.09±0.01 | 0.16±0.02 | 27.25±1.40 | 0.92±0.09 |
| *Polygonum aviculare* | 1.19±0.65 | 0.40±0.15 | 0.17±0.10 | 0.47±0.56 | 19.75±6.08 | 1.08±0.76 |
| *Taraxacum mongolicum* | 5.65±2.34 | 2.14±1.03 | 0.12±0.07 | 4.11±1.37 | 20.20±7.46 | 3.48±1.74 |
| *Tribulus terrestris* | 1.43±0.29 | 0.73±0.33 | 0.09±0.07 | 0.65±0.52 | 21.51±7.41 | 1.84±0.42 |
| *Halogeton arachnoideus* | 3.00±NA | 0.30±NA | 0.36±NA | 1.65±NA | 28.62±NA | 2.14±NA |
| *Chloris virgata* | 12.50±3.1 | 0.30±0.1 | 0.26±0.14 | 4.50±0.44 | 17.3±1.7 | 1.41±0.15 |
| *Ceratocarpus arenarius* | 1.41±2.39 | 0.27±0.10 | 0.27±0.22 | 0.26±0.40 | 16.66±6.82 | 1.37±0.81 |
| *Plantago asiatica* | 4.75±2.47 | 0.70±0.71 | 0.16±0.21 | 2.28±2.16 | 16.53±4.14 | 2.11±1.49 |
| *Stipa capillata* | 6.26±4.54 | 0.20±0.13 | 0.29±0.23 | 0.17±0.08 | 8.59±5.43 | 2.08±1.67 |
| *Artemisia gmelinii* | 0.59±0.27 | 0.11±0.06 | 0.16±0.11 | 0.09±0.08 | 18.77±7.99 | 1.47±0.28 |
| *Grubovia dasyphylla* | 0.88±0.29 | 0.30±0.09 | 0.35±0.76 | 0.19±0.07 | 17.53±7.41 | 1.71±1.17 |
| *Krascheninnikovia ceratoides* | 1.54±0.25 | 0.33±0.14 | 0.18±0.18 | 0.38±0.03 | 17.01±14.83 | 6.45±7.70 |
| *Peganum harmala* | 3.03±0.93 | 0.28±0.13 | 0.17±0.15 | 0.59±0.37 | 26.24±0.92 | 1.51±0.18 |
| *Geranium wilfordii* | 3.95±1.12 | 5.61±1.73 | 0.22±0.71 | 11.83±7.93 | 26.01±3.76 | 2.82±1.39 |
| *Euphrasia pectinata* | 1.60±0.1 | 2.05±0.05 | 0.20±0.07 | 1.55±0.05 | 25.45±1.90 | 3.75±0.09 |
| *Veronica campylopoda* | 0.40±0.1 | 0.15±0.05 | 0.14±0.05 | 0.65±0.05 | 15.51±0.07 | 3.75±0.18 |
| *Iris tectorum* | 12.30±1.70 | 0.50±0.08 | 0.65±0.05 | 5.50±0.80 | 11.60±1.45 | 1.75±0.06 |
| *Cerastium arvense* | 0.30±0.05 | 0.25±0.04 | 0.08±0.04 | 0.15±0.04 | 6.80±0.60 | 1.24±0.05 |
| *Thalictrum aquilegiifolium* | 2.48±0.53 | 2.73±1.13 | 0.28±0.11 | 4.35±2.74 | 22.09±4.18 | 1.97±0.49 |
| *Fragaria vesca* | 2.63±0.22 | 2.88±0.90 | 0.18±0.01 | 5.08±1.49 | 21.79±3.14 | 0.72±0.17 |
| *Sibbaldianthe bifurca* | 1.90±024 | 1.42±0.66 | 0.25±0.02 | 1.46±0.92 | 15.04±1.36 | 1.02±0.39 |
| *Astragalus membranaceus* | 1.68±0.18 | 0.64±0.23 | 0.28±0.06 | 1.18±0.28 | 29.14±5.00 | 2.09±0.09 |
| *Lappula myosotis* | 1.06±0.76 | 0.27±0.12 | 0.10±0.11 | 0.26±0.17 | 20.61±3.28 | 1.43±1.16 |
| *Alhagi camelorum* | 1.1±0.1 | 0.85±0.15 | 0.39±0.01 | 0.71±0.39 | 23.12±0.44 | 1.767±0.04 |
| *Bassia prostrata* | 1.4±0.3 | 0.24±0.14 | 0.35±0.02 | 0.26±0.14 | 14.60±0.53 | 1.94±0.09 |
| *Medicago falcata* | 1.35±0.15 | 0.50±0.11 | 0.26±0.01 | 0.90±0.05 | 21.11±0.04 | 1.79±0.16 |

**Seasonal dynamics of community types based on dominant family**

The composition of plant community types based on dominant family in the desert steppe of the Ili River Valley exhibited dynamic characteristics over time ([Figure S1](#FigureS1)). Species from the Asteraceae-dominated clearly dominated the study area, accounting for over 40% of the plot proportion each month and showing a consistent increasing trend over time. In contrast, species from the Chenopodiaceae-dominated and Cyperaceae-dominated displayed a similar pattern, initially increasing and then decreasing, with both peaking at 25% in July and falling below 13% in May and September. Species from the Poaceae-dominated showed a trend of initial decline followed by an increase, reaching a peak of over 18% in May and dropping to a low of less than 7% in July. Species from the Fabaceae-dominated remained relatively stable in May and July, accounting for less than 3.5%, but increased to over 15% in September. Meanwhile, species from other-dominated declined from May to July, also falling below 3.5% in July.





**Supplementary** **Figure** **1**

**Figure S1. Temporal dynamics of community types based on dominant family composition.** Temporal variations across May, July, and September, expressed as plot proportion (%). Different colors represent different community types: Others (green), Fabaceae (blue), Cyperaceae (light blue), Poaceae (yellow), Chenopodiaceae (orange), and Asteraceae (red).

**Seasonal Dynamics of Plant Diversity Among Different Community Types**

Plant diversity indices exhibited significant seasonal dynamics across different community types, with an overall trend of a notable decline in September ([Figure S2](#FigureS2)). Specifically, for the As, both the Simpson (*D*) and Pielou indices in May were significantly higher than those in September, decreasing by 42.8% (*P* < 0.05) and 33.4% (*P* < 0.05), respectively. No significant differences were observed between July and either May or September. The Shannon-Wiener index (*H*) in May was significantly higher than in July and September, decreasing by 34.3% (*P* < 0.05) and 56.4% (*P* < 0.01), respectively. Additionally, the ln*S* index in September was significantly lower than in May and July, declining by 53.7% (*P* < 0.01) and 43.6% (*P* < 0.05), respectively. In the Ch, only the ln*S* index showed a significant decrease of 38.1% (*P* < 0.05) in September compared to May, while the other indices exhibited no significant monthly variations. For the Po, no significant differences were detected in the Simpson (*D*), Shannon-Wiener (*H*), or Pielou indices. However, the ln*S* index in September was significantly lower than in May and July, with reductions of 51.5% (*P* < 0.01) and 50.5% (*P* < 0.01), respectively.Similarly, in the Cy, the ln*S* index in September was significantly lower than in May and July, declining by 62.4% (*P* < 0.05) and 66.8% (*P* < 0.05), respectively. As for the Fa, the Simpson and Shannon-Wiener indices in September decreased significantly by 56.2% (*P* < 0.05) and 65.7% (*P* < 0.05), respectively, compared to July. The ln*S* index also showed a significant reduction of 67.5% (*P* < 0.05) in September, while the Pielou index remained unchanged. Furthermore, in the Ot, none of the diversity indices exhibited significant differences between May and July.





**Supplementary** **Figure 2**

**Figure S2.** Violin Plots Illustrating Temporal Dynamics of Biodiversity Indices Among Plant community types and Months. Violin plots display the distributions of four biodiversity indices—Simpson (*D*) index, Shannon-Wiener (*H*) index, Pielou index, and ln*S* index—across three months (May, July, and September, coded as green, blue, and orange, respectively) and six plant community types: As (Asteraceae-dominated), Ch (Chenopodiaceae-dominated), Po (Poaceae-dominated), Cy (Cyperaceae-dominated), Fa (Fabaceae) and Ot (Other families-dominated). Different lowercase letters denote significant differences between months (*P* < 0.05).

**Relationships between elevation and plant functional traits across seasons**

From a seasonal dynamics perspective, as July spans the 1000–2500 m elevation range, plant functional traits gradually increase with elevation ([Figure S3](#FigureS3)). Leaf length, leaf width, and leaf area all showed significant positive correlations with elevation (*P* < 0.001), while leaf thickness also exhibited a positive correlation (*P* = 0.005). Conversely, in May and September, due to incomplete elevation gradients, no significant correlations were found between plant functional traits and elevation.





**Supplementary Figure 3**

**Figure S3. Relationships between elevation and plant functional traits across seasons.** Scatter plots showing the relationships between elevation (m) and leaf traits (length, width, thickness, area) in May (A, D, G, J), July (B, E, H, K), and September (C, F, I, L). Each data point represents the mean trait value for each species within a single plot, weighted by species importance value for analysis. Different colors denote distinct sampling months (blue dots indicate May, green dots indicate July, and orange dots indicate September). *R²_m_* (marginal *R²*) and *R²_C_* (conditional *R²*) were employed to assess model fit, with *P*-values indicating the significance level of regression relationships.

**Seasonal dynamics of plant diversity and spatial heterogeneity**

The spatial heterogeneity of plant diversity indices (Simpson, Shannon-Wiener, Pielou, and ln*S*) within the study area exhibited dynamic variations ([Figure S4](#FigureS4)). Overall, plant diversity displayed a patchy mosaic pattern with pronounced spatial heterogeneity. High-value areas were primarily concentrated in Nilek, with these zones extending in a gradient toward its periphery, while low-value areas were mainly clustered in Xinyuan and Yining. Seasonal dynamics revealed that May and July were periods of pronounced spatial heterogeneity, with central areas forming core high-value zones for plant diversity. Notably, however, ln*S* values were low across nearly all surveyed areas in September, which contrasted with the high values observed around Huocheng in July.


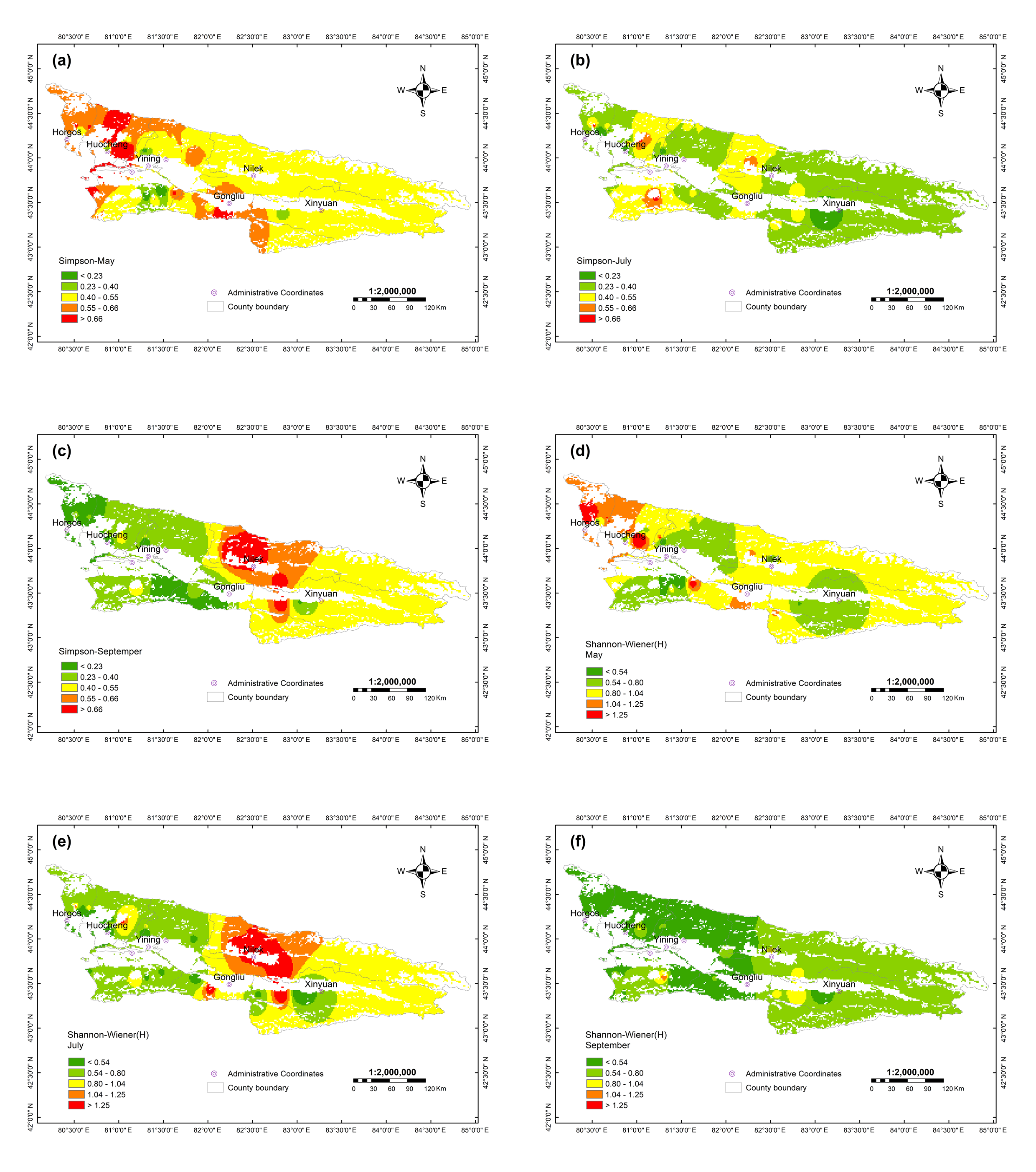


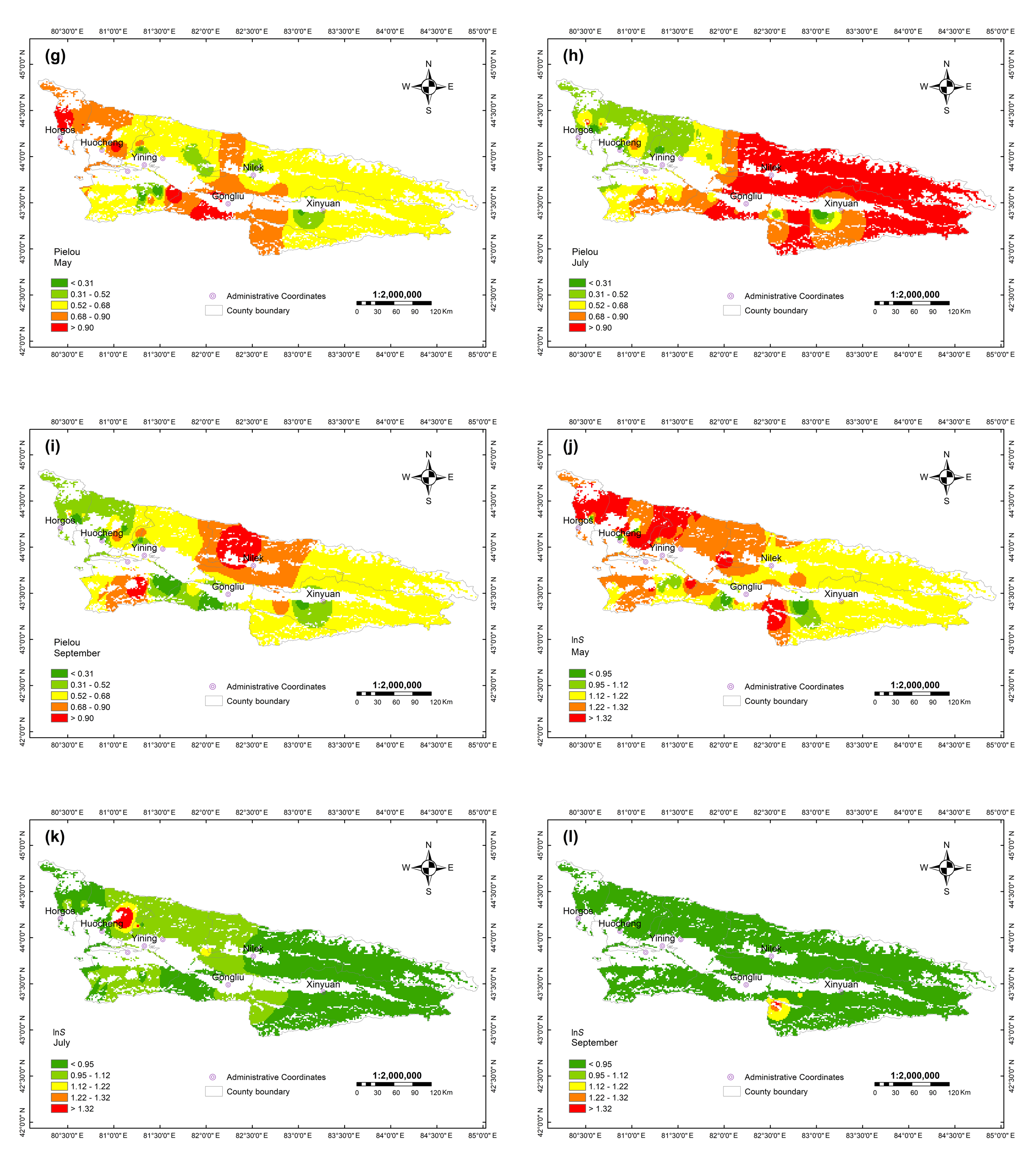


**Supplementary Figure 4**

**Figure S4. Spatial patterns of plant diversity indices.** These figures show the spatial distribution of Simpson, Shannon-Wiener, Pielou, and ln*S* indices across three sampling periods (May, July, September). Different colors represent significant differences among vegetation types. The color gradient from green to red represents the range from low to high values of each index. Administrative Coordinates represent the locations of counties (Horgos, Huocheng, Yining, Nilek, Gongliu, Xinyuan), and County Boundary represents the county administrative boundaries. The scale is 1:2,000,000.

**Seasonal patterns in plant functional traits and spatial heterogeneity**

Research findings revealed that spatial heterogeneity in leaf functional traits (leaf length, leaf width, leaf thickness, and leaf area) exhibited dynamic characteristics ([Figure S5](#FigureS5)): Overall, high-value zones for plant functional traits were primarily concentrated in Nilek and Huocheng, while low-value zones—except for leaf thickness, which was mainly concentrated in Horgos at a low of 0.06 mm—were predominantly found in Gongliu and Xinyuan. From a seasonal dynamics perspective, July represented a period of pronounced spatial heterogeneity, with plant traits exhibiting distinct patchy distributions. High-value zones for leaf length, leaf width, and leaf area were concentrated around Yining and Nilek, while high-value zones for leaf thickness were primarily found in Xinyuan. Notably, May showed low values for leaf length and leaf thickness across the entire study area, and September exhibited low leaf area values throughout the region. Additionally, leaf thickness exhibited high clustering in September's high-value zones, with low-value areas appearing only as scattered points.


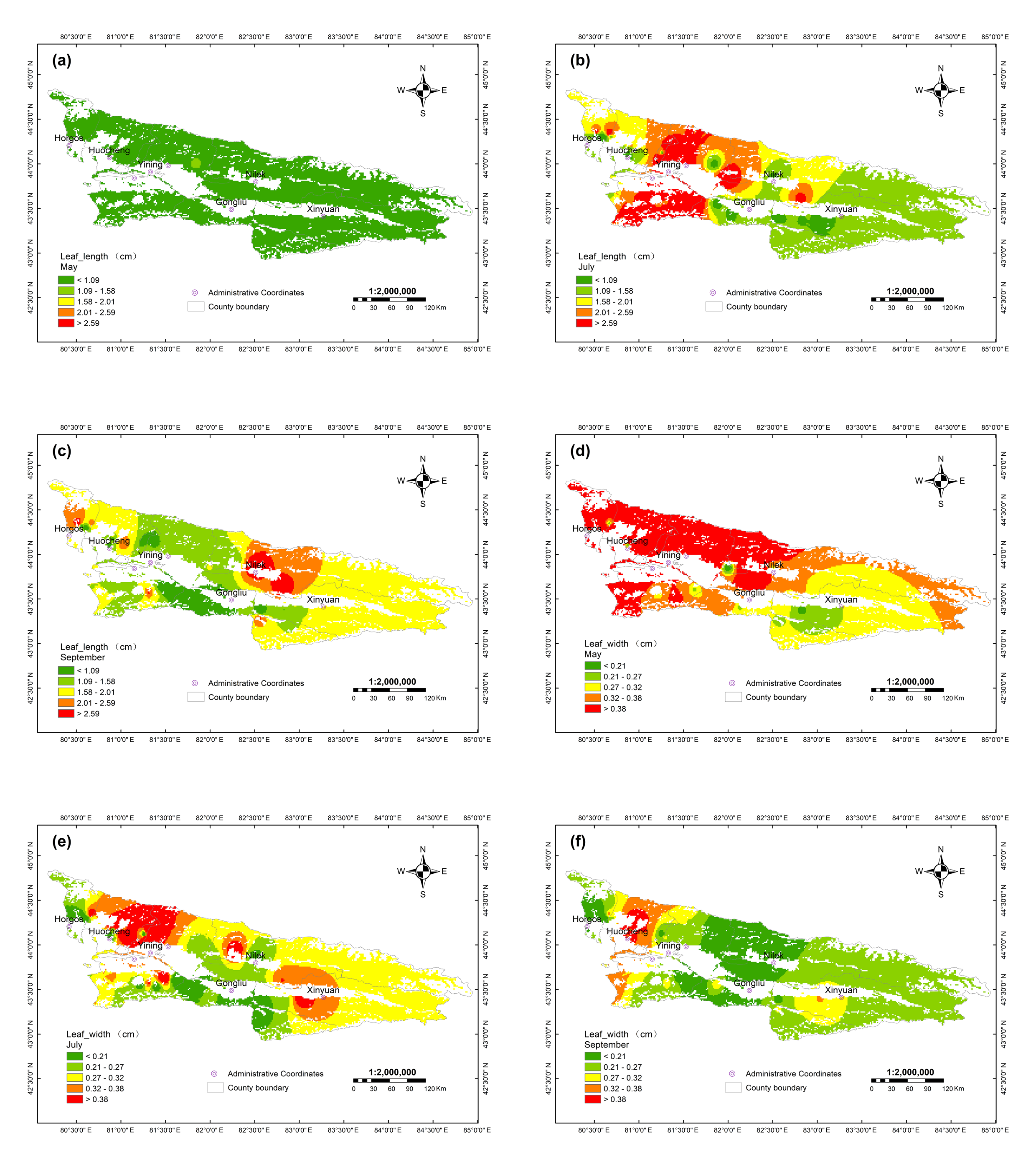


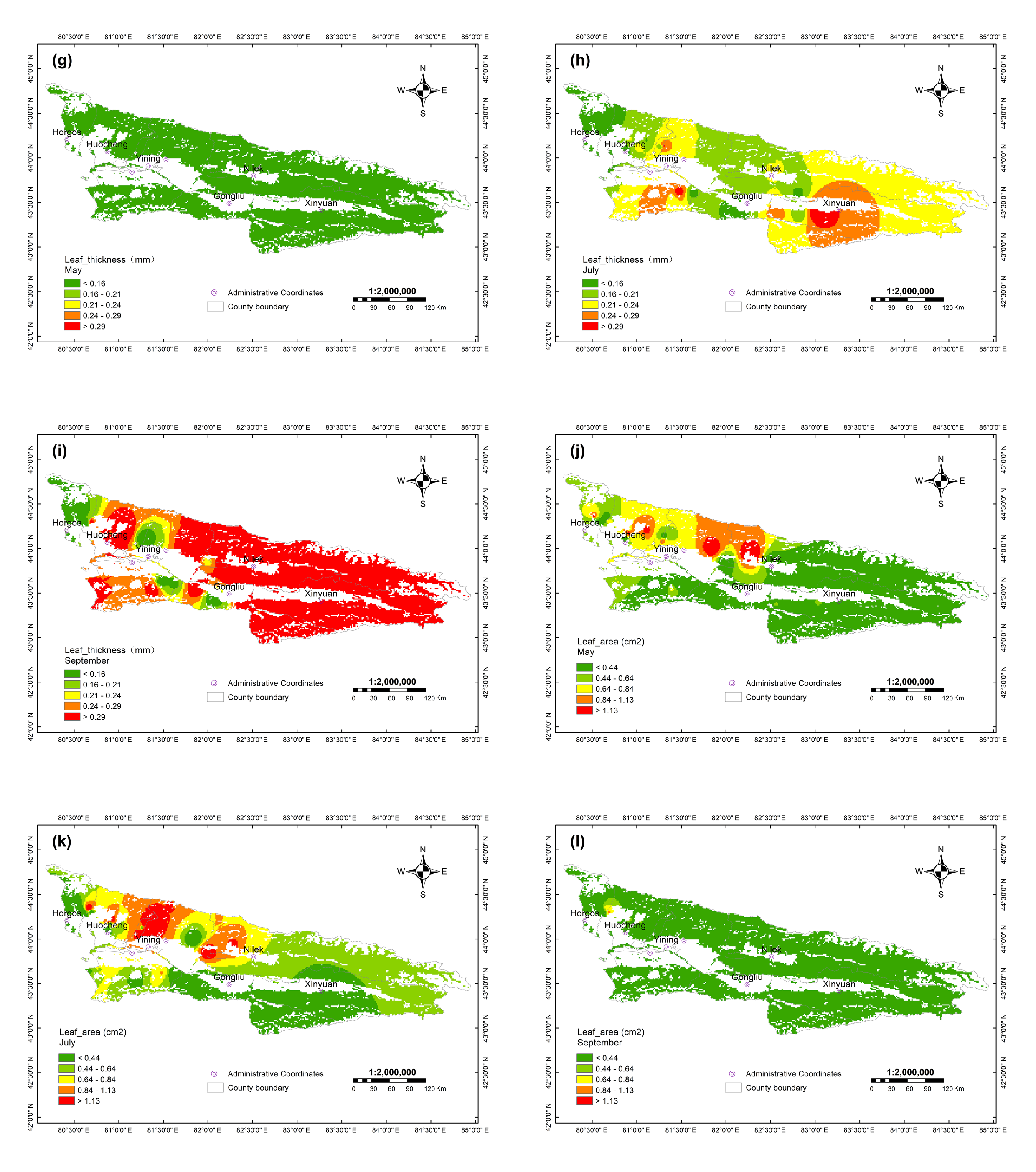


**Supplementary Figure 5**

**Figure S5. Spatial patterns of plant functional traits.** These figures illustrate the spatial distribution of leaf length (cm), leaf width (cm), leaf thickness (mm), and leaf area (cm²) across three sampling periods (May, July, September). Different colors represent significant differences among vegetation types. The color gradient from green to red represents the range from low to high values of each index. Administrative Coordinates represent the locations of counties (Horgos, Huocheng, Yining, Nilek, Gongliu, Xinyuan), and County Boundary represents the county administrative boundaries. The scale is 1:2,000,000.

**Seasonal Variation in Plant Functional Traits**

Overall, leaf length, width and leaf area all reached their highest values in July ([Figure S6](#FigureS6)). Specifically, leaf length in September decreased by 45.56% and 50.68% compared to May and July respectively, representing a significant difference (*P* < 0.01). Leaf width decreased by 40.92% and 67.27% in May and September respectively compared to July, representing significant differences (*P* < 0.01). Similarly, leaf area decreased by 64.51% and 81.42% in May and September respectively compared to July (*P* < 0.01). However, leaf thickness was lower in May and July than in September, decreasing by 79.70% and 22.61% respectively (*P* < 0.01). Concurrently, leaf nitrogen content decreased by 12% and 20.72% in September and July respectively compared to May (*P* < 0.01). Conversely, leaf phosphorus content increased by 34.2% in September compared to May (*P* < 0.01) and by 19.12% compared to July (*P* < 0.05).





**Supplementary Figure 6**

**Figure S6. Seasonal variations in leaf functional traits of desert steppe plants from May to September.** Different lowercase letters above the bars indicate significant differences among months.

**Patterns of Functional Traits in Leaves Along an Elevational Gradient under Seasonal Dynamics**

This figure (A–F) illustrates the variation in functional leaf traits of desert steppe plants with altitude (750–1500 m). Results from mixed linear models indicate that the marginal *R²* (*R²m*) for all traits was close to zero, with *P*-values exceeding 0.05. This suggests that across the studied altitudinal gradient, the community-weighted mean of leaf functional traits did not exhibit a significant linear trend.


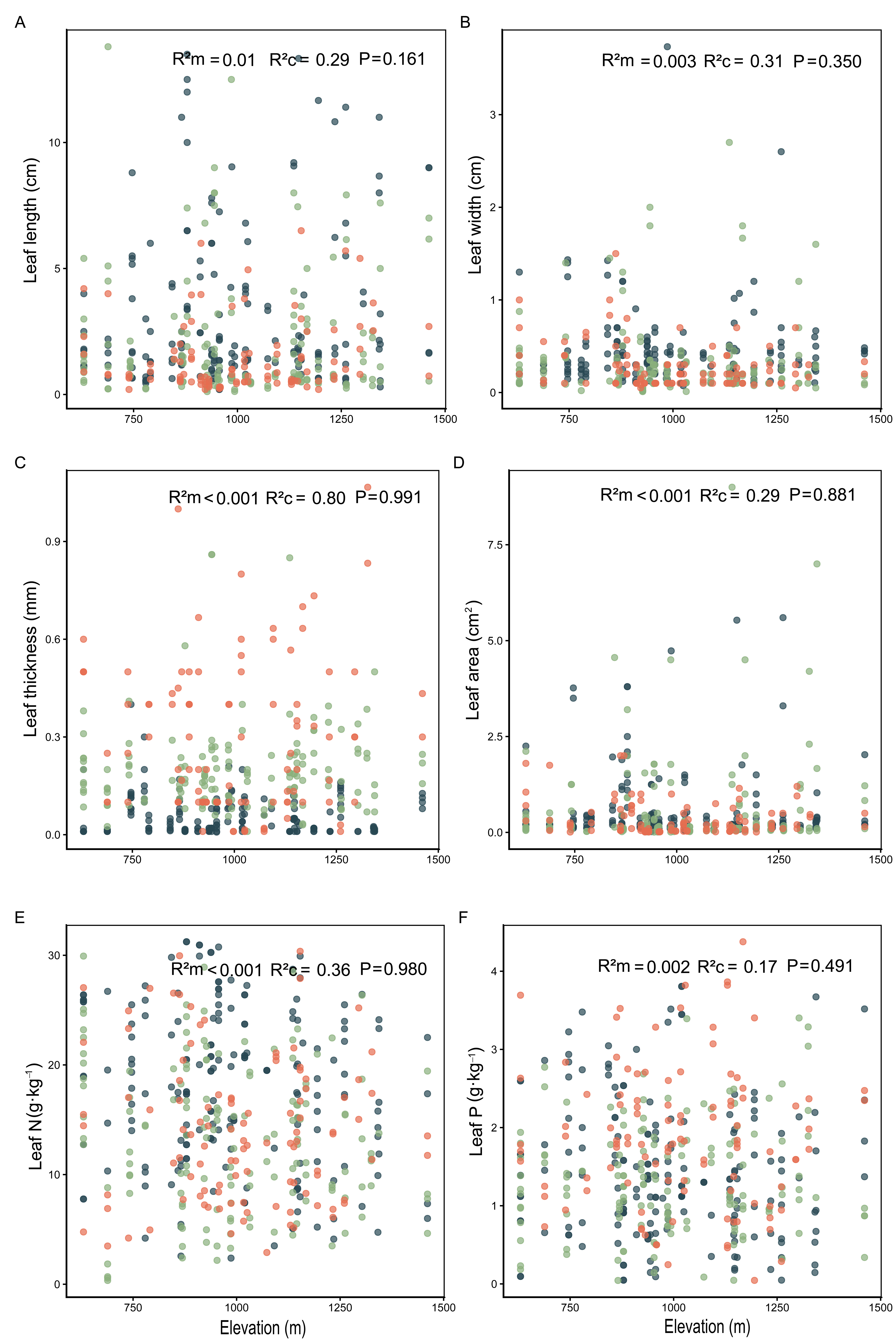


**Supplementary Figure 7**

**Figure S7. Changes in functional traits of desert steppe plant leaves along an elevational gradient under seasonal dynamics.** (A) Leaf length (cm); (B) Leaf width (cm); (C) Leaf thickness (mm); (D) Leaf area (cm^2^); (E) Leaf nitrogen content (g·kg^-1^); (F) Leaf phosphorus content (g·kg^-1^). Each data point represents the mean trait value for each species within a single plot, weighted by species importance value for analysis. Different colors denote distinct sampling months (blue dots indicate May, green dots indicate July, and orange dots indicate September). *R²m* (marginal *R²*) and *R²c* (conditional *R²*) were employed to assess model fit, with *P*-values indicating the significance level of regression relationships.


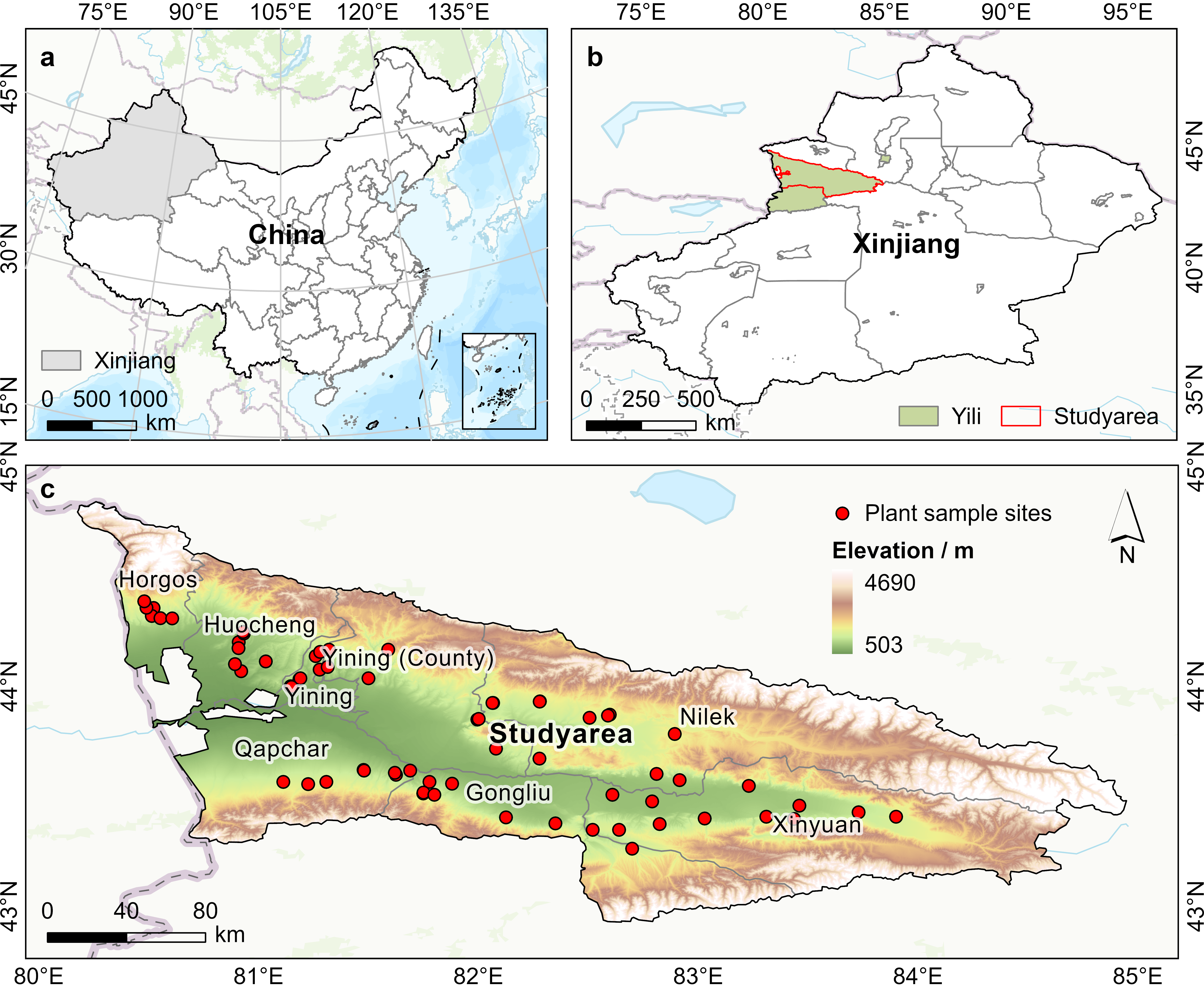


**Supplementary Figure 8**

**Figure S8. Distribution of research areas and plant sampling points in July.** (a) China; (b) Xinjiang; and (c) Study area. Digital elevation model (DEM) of the study area, illustrating the spatial distribution of plant sampling sites (red dots). Visualised based on the standard map (Review Number: GS(2024)0650) obtained from the National Geographic Information Public Service Platform of China.

**Elevation and Seasonal Interactions Drive Variation in Plant Nitrogen and Phosphorus Content**

From a seasonal dynamics perspective, plant functional traits in July showed a gradual increasing trend with elevation. Plant Nitrogen and Phosphorus Content all exhibited significant positive correlations with elevation (*P* < 0.001), covering a broad elevation range of 1000–2500 m. In contrast, in May and September, which was primarily distributed in the lower elevation range of 750–1500 m, no significant correlation was found between plant functional traits and elevation.


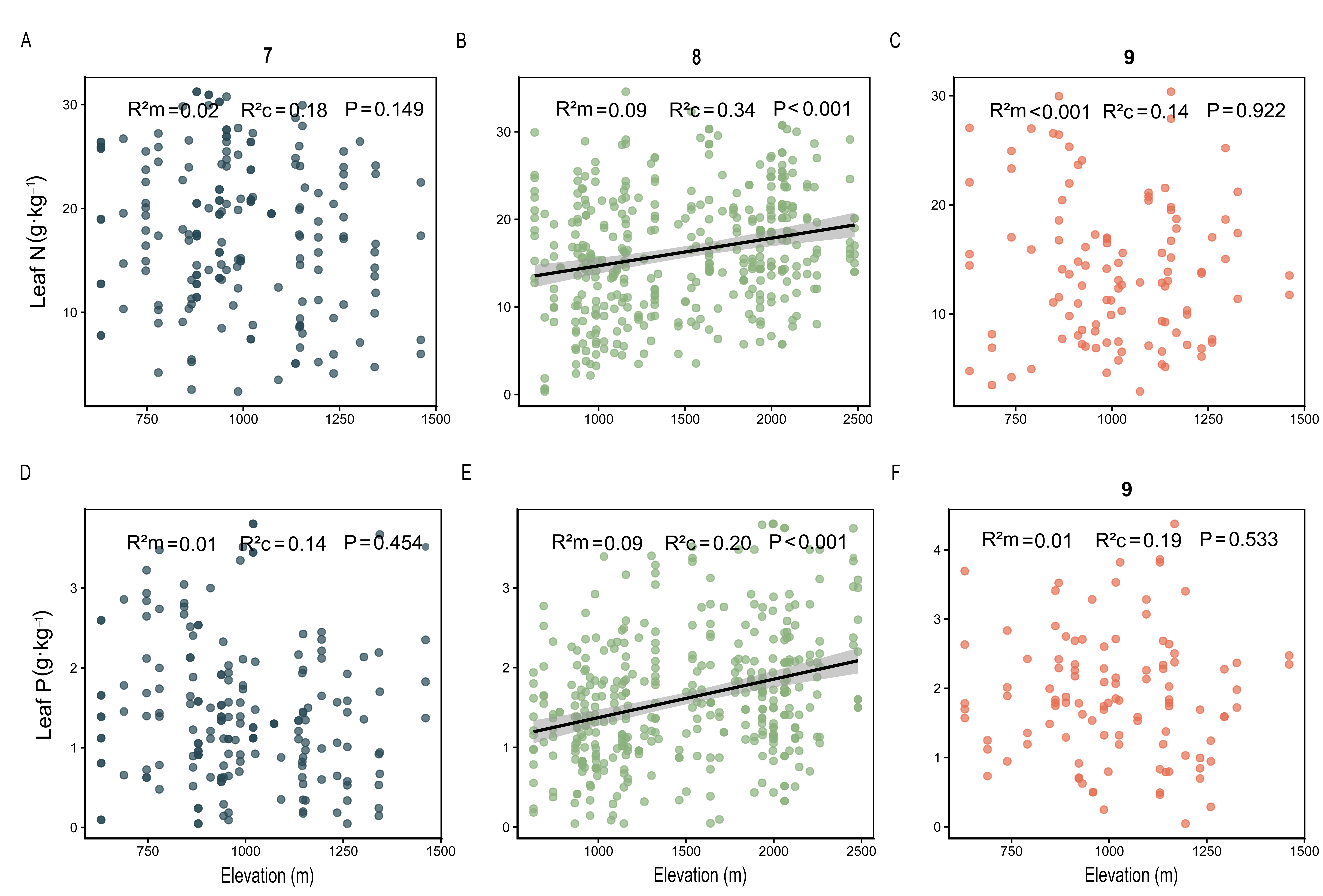


**Supplementary Figure 9**

**Figure S9. Elevation and Seasonal Interactions Drive Variation in Plant Nitrogen and Phosphorus Content.** (A-C) Leaf nitrogen content (g·kg^-1^); (D-F) Leaf phosphorus content (g·kg^-1^). Each data point represents the mean trait value for each species within a single plot, weighted by species importance value for analysis. Different colors denote distinct sampling months (blue dots indicate May, green dots indicate July, and orange dots indicate September). *R²m* (marginal *R²*) and *R²c* (conditional *R²*) were employed to assess model fit, with *P*-values indicating the significance level of regression relationships.
